# Peroxisomal Fatty Acid Oxidation and Glycolysis Are Triggered in Mouse Models of Lesional Atopic Dermatitis

**DOI:** 10.1016/j.xjidi.2021.100033

**Published:** 2021-06-15

**Authors:** Petra Pavel, Géraldine Leman, Martin Hermann, Christian Ploner, Thomas O. Eichmann, Deborah Minzaghi, Franz P.W. Radner, Barbara Del Frari, Robert Gruber, Sandrine Dubrac

**Affiliations:** 1 Department of Dermatology, Venereology and Allergology, Medical University of Innsbruck, Innsbruck, Austria; 2 KMT Laboratory, Department of Visceral, Transplant and Thoracic Surgery, Center for Operative Medicine, Medical University of Innsbruck, Innsbruck, Austria; 3 Department of Plastic, Reconstructive and Aesthetic Surgery, Medical University of Innsbruck, Innsbruck, Austria; 4 Institute of Molecular Biosciences, University of Graz, Graz, Austria

**Keywords:** AD, atopic dermatitis, ADL, lesional atopic dermatitis, ATP, adenosine triphosphate, Cer, ceramide, CoA, coenzyme A, FA, fatty acid, FFA, free fatty acid, HEE, human epidermal equivalent, IMQ, imiquimod, KC, keratinocyte, KO, knockout, LB, lamellar body, PPAR, peroxisome proliferator–activated receptor, SC, stratum corneum, TEWL, transepidermal water loss, ULCFA, ultra long-chain fatty acid, VLCFA, very-long-chain fatty acid

## Abstract

Alterations of the lipid profile of the stratum corneum have an important role in the pathogenesis of atopic dermatitis (AD) because they contribute to epidermal barrier impairment. However, they have not previously been envisioned as a cellular response to altered metabolic requirements in AD epidermis. In this study, we report that the lipid composition in the epidermis of flaky tail, that is, *ft/ft* mice mimics that of human lesional AD (ADL) epidermis, both showing a shift toward shorter lipid species. The amounts of C_24_ and C_26_ free fatty acids and C_24_ and C_26_ ceramides—oxidized exclusively in peroxisomes—were reduced in the epidermis of *ft/ft* mice despite increased lipid synthesis, similar to that seen in human ADL edpidermis. Increased ACOX1 protein and activity in granular keratinocytes of *ft/ft* epidermis, altered lipid profile in human epidermal equivalents overexpressing ACOX1, and increased ACOX1 immunostaining in skin biopsies from patients with ADL suggest that peroxisomal β-oxidation significantly contributes to lipid signature in ADL epidermis. Moreover, we show that increased anaerobic glycolysis in *ft/ft* mouse epidermis is essential for keratinocyte proliferation and adenosine triphosphate synthesis but does not contribute to local inflammation. Thus, this work evidenced a metabolic shift toward enhanced peroxisomal β-oxidation and anaerobic glycolysis in ADL epidermis.

## Introduction

Atopic dermatitis (AD) is a chronic and relapsing inflammatory skin disorder with a high prevalence worldwide. Although associations with genetic and environmental factors have been established, AD pathogenesis is still not fully understood (Czarnowicki et al., 2017; Guttman-Yassky et al., 2017; Leung and Guttman-Yassky, 2014). Nevertheless, a defective epidermal barrier is likely involved in disease initiation (Kelleher et al., 2015). Indeed, this defect is hypothesized to make the skin more vulnerable to environmental triggers such as allergens, microbes, or pollutants, which in turn elicit a local and humoral atopic immune response (Leung, 2016; Leung and Guttman-Yassky, 2014). Compensatory responses to the barrier impairment include keratinocyte (KC) hyperproliferation requiring lipid, DNA, and protein synthesis, which are all energy-intensive processes (Ajani et al., 2007; Blunder et al., 2018; Proksch et al., 1993).

In AD, an overall decline in lipid content in the skin has been documented (Angelova-Fischer et al., 2011; Elias and Wakefield, 2014; Proksch et al., 2003; Schäfer and Kragballe, 1991) in association with dysregulated expression of genes controlling epidermal lipid metabolism (Blunder et al., 2018; Cole et al., 2014). Moreover, there is a clear shift toward shorter chain fatty acid (FA) and ceramide (Cer) species in the stratum corneum (SC) of patients with AD (Berdyshev et al., 2018; Ishikawa et al., 2010; Janssens et al., 2012; van Smeden et al., 2014b), which contributes to aberrant lipid organization of the lamellar bilayers (Janssens et al., 2012). This is consistent with the altered content and secretion of lamellar bodies (LBs) observed in AD epidermis (Blunder et al., 2017; Elias and Wakefield, 2014). However, these changes in lipid metabolism have not previously been envisioned as a cellular response to altered metabolic requirements in AD epidermis. To date, a thorough understanding of lipid and energy metabolism in KCs in AD is still missing.

Previous work has shown that the dermal vasculature can supply the epidermis with nutrients, including glucose and FAs (Gherzi et al., 1992; Grubauer et al., 1987; Khnykin et al., 2011; Zhang et al., 2018; Ziboh et al., 1986). However, the mode of energy metabolism used by KCs to fulfill their bioenergetic and biosynthetic needs in AD is still unknown. In this work, we investigated glycolysis and FA metabolism in lesional AD (ADL) epidermis. Moreover, we sought to determine whether the metabolic changes are a cause or a consequence of the disease.

## Results

### Lipid composition in flaky tail mouse epidermis mimics that in human ADL epidermis

**Figure 1:**
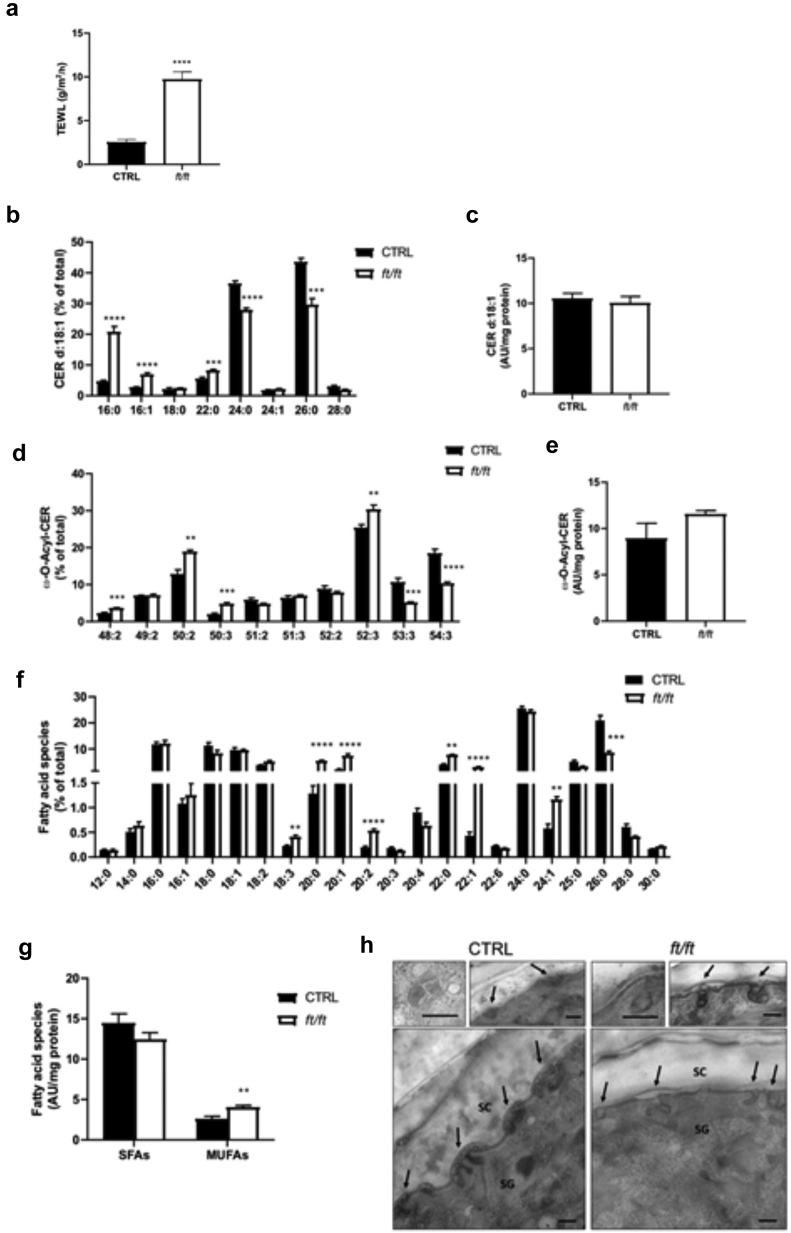
**Lipid composition and ultrastructural analysis of *ft/ft* mouse epidermis.** (**a**) The function of the inside‒out epidermal barrier was assessed by measuring TEWL on the ears of control and *ft/ft* mice (n = 5). Relative and total amounts of (**b, c**) d18:1 Cers(NS), (**d**, **e**) ω-O-acylCers (n = 5), (**f**, **g**) SFAs and MUFAs in the epidermis of *ft/ft* mice compared with those in the epidermis of control mice (n = 5). Data are shown as analyte/IS ratio (AU) per mg protein or as the relative percentage of total lipid species. (**h**) Ultrastructural analysis of control (left) and *ft/ft* (right) mouse epidermis showing LB morphology (insets) and secretion (arrows). Osmium tetroxide after fixation. Bar = 250 nm. Data were analyzed with a Student’s *t*-test. ∗∗*P* < 0.01, ∗∗∗*P* < 0.001, and ∗∗∗∗*P* < 0.0001. AU, arbitrary unit; Cer, ceramide; CTRL, control; IS, internal standard; LB, lamellar body; MUFA, monounsaturated fatty acid; SC, stratum corneum; SFA, saturated fatty acid; SG, stratum granulosum; TEWL, transepidermal water loss.

Flaky tail (*ft/ft*) mice are a model of ADL that harbor two gene mutations, that are, mutations of *Flg* and *Tmem79* (Kypriotou et al., 2013; Moniaga et al., 2010; Sasaki et al., 2013; Saunders et al., 2013). The skin of *ft/ft* mice displays impaired permeability barrier function, as measured by increased transepidermal water loss (TEWL) (Figure 1a) (Moniaga et al., 2010; Saunders et al., 2013), which is also observed in human ADL skin regardless of disease severity, in contrast to nonlesional AD skin (Seidenari and Giusti, 1995; Yazdanparast et al., 2019). To validate *ft/ft* mice as a model of ADL with respect to lipid composition, we first studied the chain length distribution in (i) epidermal Cers containing a C_18_ sphingosine base linked to a nonhydroxy FA (i.e., Cer[NS]), (ii) free FAs (FFAs), and (iii) very long chain, ester-linked ω-O-acylCers (i.e., Cer[EO]). We found that *ft/ft* mouse epidermis has a marked increase in the relative levels of Cer(NS)-containing C_16_–C_22_ FA moieties and a decrease in those harboring C_24_ and C_26_ very-long-chain fatty acid (VLCFA) moieties, whereas total Cer(NS) content remained unchanged (Figure 1b and c). Similarly, ω-O-acylCers contained diminished levels of 53:3 and 54:3 species and increased levels of several species with shorter chain lengths, without significant changes in the total amounts (Figure 1d and e). Thus, our data reveal alterations in Cer classes in the epidermis of *ft/ft* mice similar to those described in human ADL (Berdyshev et al., 2018; Ishikawa et al., 2010; Janssens et al., 2012; van Smeden et al., 2014b). Moreover, the relative amounts of saturated FAs with 20–22 carbon atoms were higher and amounts of those with 26 carbon atoms were lower in the epidermis of *ft/ft* mice than in the epidermis of control mice (Figure 1f). Furthermore, consistent with previous observations on SC or epidermal specimens of ADL (Schäfer and Kragballe, 1991; van Smeden et al., 2014b), we found higher total levels of monounsaturated FAs in the epidermis of *ft/ft* mice than those in the epidermis of control mice, in contrast to the total levels of saturated FAs (Figure 1g). Shortening of the FA moiety in Cers has been shown to alter the formation of SC lamellar bilayers, a process initiated in LBs (Feingold and Elias, 2014; Janssens et al., 2012; van Smeden et al., 2014b). Ultrastructural analysis showed LBs to be scarce, empty, or filled with inhomogeneous material and to exhibit altered morphology in the epidermis of *ft/ft* mice, indicating abnormal cargo composition and altered lamellar bilayers in the SC (Figure 1h), as reported earlier (Scharschmidt et al., 2009). Thus, abnormalities of epidermal lipid composition and of the LB secretory system in *ft/ft* mice largely mimic those observed in human ADL (Berdyshev et al., 2018; Blunder et al., 2017; Elias and Wakefield, 2014; Ishikawa et al., 2010; Janssens et al., 2012; van Smeden et al., 2014b).

### Peroxisomal FA oxidation is enhanced in *ft/ft* mouse epidermis

**Figure 2:**
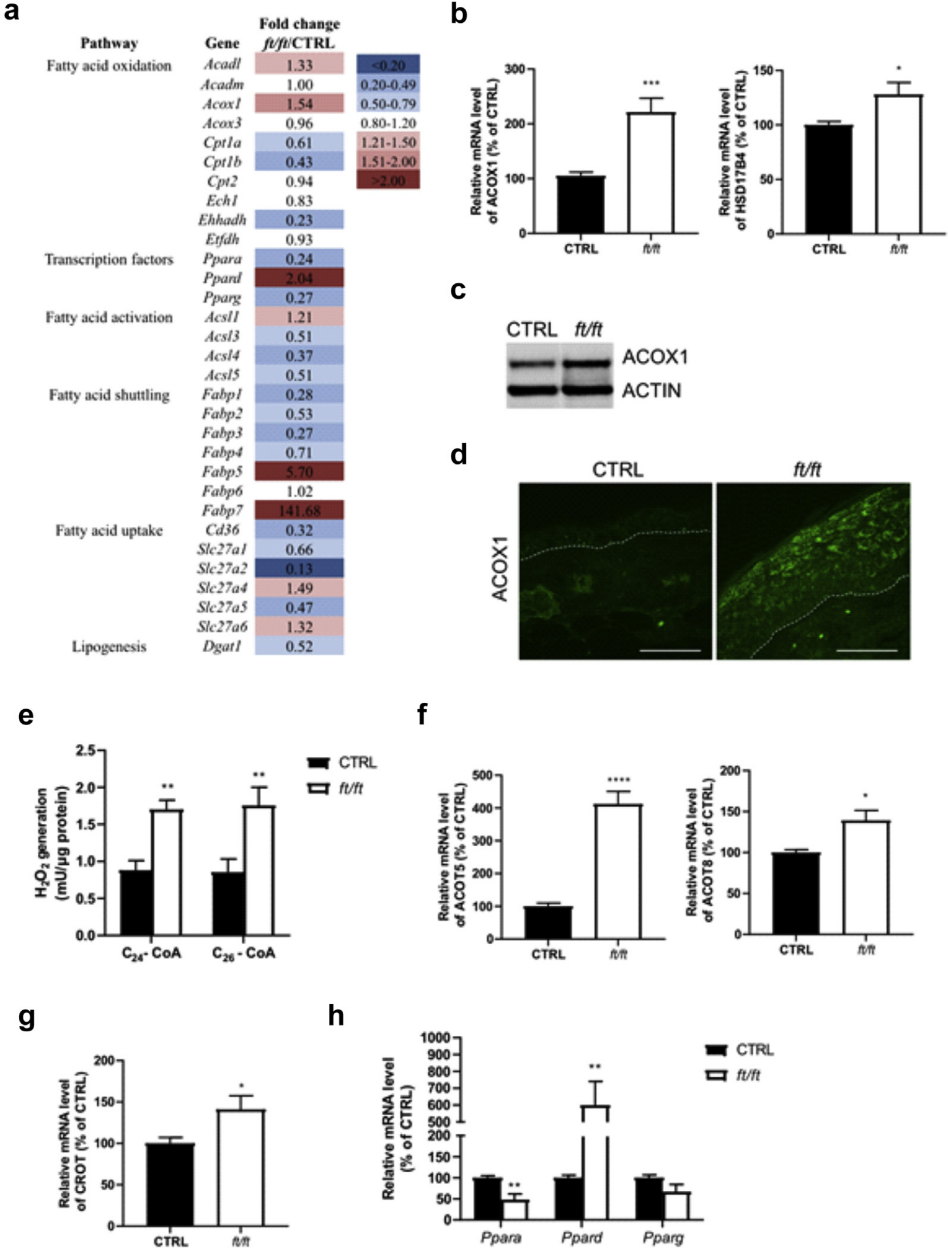
**Changes in lipid metabolism in *ft/ft* mouse epidermis.** (**a**) Microarray analysis showing a subset of genes involved in FA metabolism in mouse epidermis. The full gene list and respective fold changes are provided in Table 3. (**b**) Relative mRNA level of *Acox1* and *Hsd17b4* in the epidermis of mice (n = 9–10). (**c, d**) Protein abundance of ACOX1 in mouse epidermis. The dashed line indicates the dermal‒epidermal boundary. Bar = 50 μm (**e**) ACOX activity measured in mouse epidermal cells (expressed as mU per μg of proteins, n = 5–7). Relative mRNA expression of (**f**) *Acot5*, *Acot8*, (**g**) *Crot*, and (**h**) PPAR mRNA, *Ppar*, isoforms in epidermal samples from CTRL and *ft/ft* mice (n = 9–10). Data were analyzed with a Student’s *t*-test. ∗*P* < 0.05, ∗∗*P* < 0.01, ∗∗∗*P* < 0.001, and ∗∗∗∗*P* < 0.0001. CTRL, control; FA, fatty acid; H_2_O_2_, hydrogen peroxide; PPAR, peroxisome proliferator–activated receptor.

To better understand the origin of the abnormal lipid composition in ADL, we examined the expression of 86 genes involved in lipid metabolism using microarray analysis. We found that genes involved in long-chain FA oxidation, activation, and trafficking were among the most strongly upregulated in the epidermis of *ft/ft* mice when compared with the epidermis of control mice (Figure 2a). In contrast, the expression of several genes involved in long-chain FA import into cells (*Fabp3*, *Slc27a1*, *Slc27a2*, *Slc27a5*, *Cd36*, *Acsl3*), and peroxisome proliferator–activated receptor (PPAR) signaling (e.g., *Ppara*, *Pparg*, *Cpt1a*, *Apoc3*, *Apoe*) was downregulated in the epidermis of *ft/ft* mice when compared with the epidermis of control mice (Figure 2a). qPCR confirmed increased mRNA level of *Acox1* (an enzyme catalyzing the oxidation of straight-chain VLCFAs and ultra long-chain FAs [ULCFAs] in peroxisomes) and of *Hsd17b4* (an enzyme participating in the second step of peroxisomal VLCFA oxidation [Baes et al., 2000]) in the epidermis of *ft/ft* mice when compared with the epidermis of the controls (Figure 2b). Moreover, ACOX1 was induced at the protein level, mainly in the upper spinous and granular layers of *ft/ft* mouse epidermis (Figure 2c and d). Furthermore, we measured ACOX enzymatic activity by following the production rate of hydrogen peroxide after the addition of very-long-chain fatty acyl-coenzyme As (CoAs) (Vamecq, 1990). We used lignoceroyl-CoA (C_24:0_-CoA) and hexacosanoyl-CoA (C_26:0_-CoA), which are VLCFAs exclusively oxidized in peroxisomes (Reddy and Hashimoto, 2001; Rizzo et al., 2003; Singh et al., 1984). Results revealed a significant increase in the oxidation of both fatty acyl-CoA species in epidermal bulk cells of *ft/ft* mice compared with that in the epidermal bluk cells of the controls (Figure 2e). Thus, peroxisomal oxidation of VLCFAs is enhanced in *ft/ft* mouse epidermis. In line with this observation, we found increased expression levels of *Acot5* and *Acot8* (Figure 2f), genes encoding two acyl-CoA thioesterases involved in downstream steps of peroxisomal FA oxidation (Westin et al., 2005), as well as the expression level of *Crot*, encoding a carnitine octanoyltransferase responsible for the transport of shortened FAs out of peroxisomes (Figure 2g) (Wanders et al., 2016; Westin et al., 2008). Thus, in a context where FA uptake is not coordinated to sustained peroxisomal lipid oxidation, VLCFAs/ULCFAs might not be sufficiently packed into LBs and integrated into structural lipids such as Cers, thereby potentially explaining the shift toward the shorter acyl chain lengths of Cers and FFAs in *ft/ft* mouse epidermis.

### PPARδ signaling is triggered in *ft/ft* mouse epidermis

**Figure 3:**
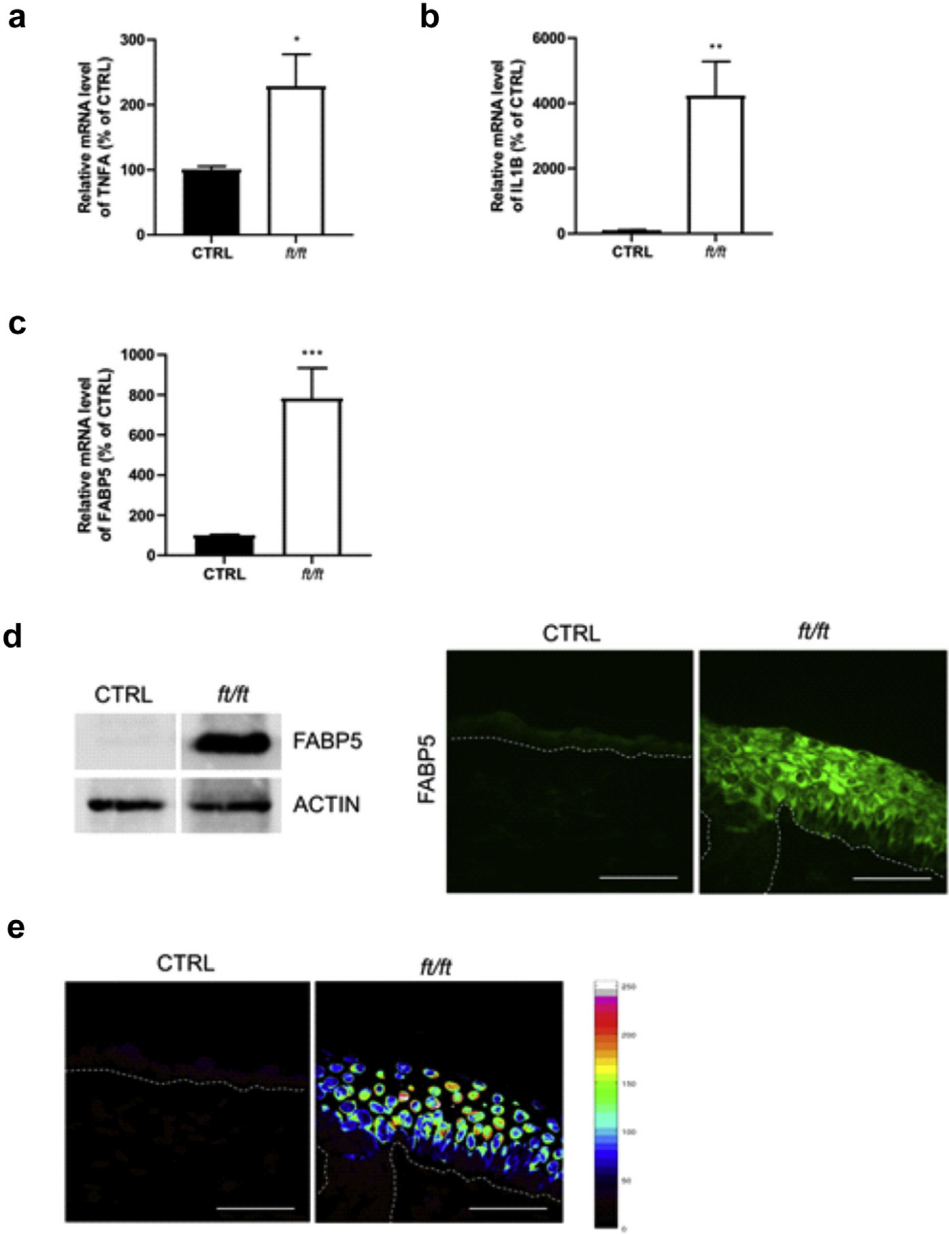
**Enhanced PPARδ signaling in *ft/ft* mouse epidermis.** Relative mRNA levels of (**a**) *Tnfa*, (**b**) *Il1b,* and (**c**) *Fabp5* in CTRL and *ft/ft* mouse epidermis (n = 9–10). (**d**) Representative western blot and immunofluorescence staining showing FABP5 in the epidermis of *ft/ft* and CTRL mice. (**e**) Nuclear localization of FAPB5 protein in mouse epidermis. The mean fluorescence intensities of FABP5 located in the nuclei are shown and represented by the spectrum of pseudocolors, ascending from black to white (see Materials and Methods). The dashed line indicates the dermal‒epidermal boundary. Bar = 50 μm. Data were analyzed with a Student’s *t*-test. ∗*P* < 0.05, ∗∗*P* < 0.01, and ∗∗∗*P* < 0.001. CTRL, control; PPAR, peroxisome proliferator–activated receptor.

PPARs are master regulators of lipid homeostasis, and reduced levels of *PPARA* and *PPARG* have been consistently observed in ADL (Plager et al., 2007; Töröcsik et al., 2019), whereas data on *PPARD* have not been reported. We found selective upregulation of *Ppard* expression in *ft/ft* mouse epidermis compared with that in control epidermis, as opposed to the expression of *Ppara* and *Pparg* (Figure 2a and h). In mouse KCs, *Ppard* expression has been shown to be upregulated by TNF-α (Michalik et al., 2003) and IL-1β (Blunder et al., 2021), cytokines increased in the epidermis of *ft/ft* mice (Figure 3a and b), likely in response to the impaired epidermal barrier (Tsai et al., 1994; Wood et al., 1992). *FABP5* has been shown to translocate to the nucleus to deliver ligands specifically to PPARδ and to be a PPARδ target gene (Schug et al., 2008; Tan et al., 2002). FABP5 was increased at both protein and mRNA levels (Figure 3c and d) and mainly localized to suprabasal KC nuclei in the epidermis of *ft/ft* mice compared with those in the epidermis of the controls (Figure 3e). Thus, enhanced PPARδ signaling might promote peroxisomal oxidation of VLCFAs/ULCFAs in *ft/ft* mouse epidermis by the upregulation of ACOX1 (Higgins et al., 2012; Luquet et al., 2005).

### Overexpression of ACOX1 disrupts the LB secretory system and enhances KC proliferation in human epidermal equivalents

**Figure 4:**
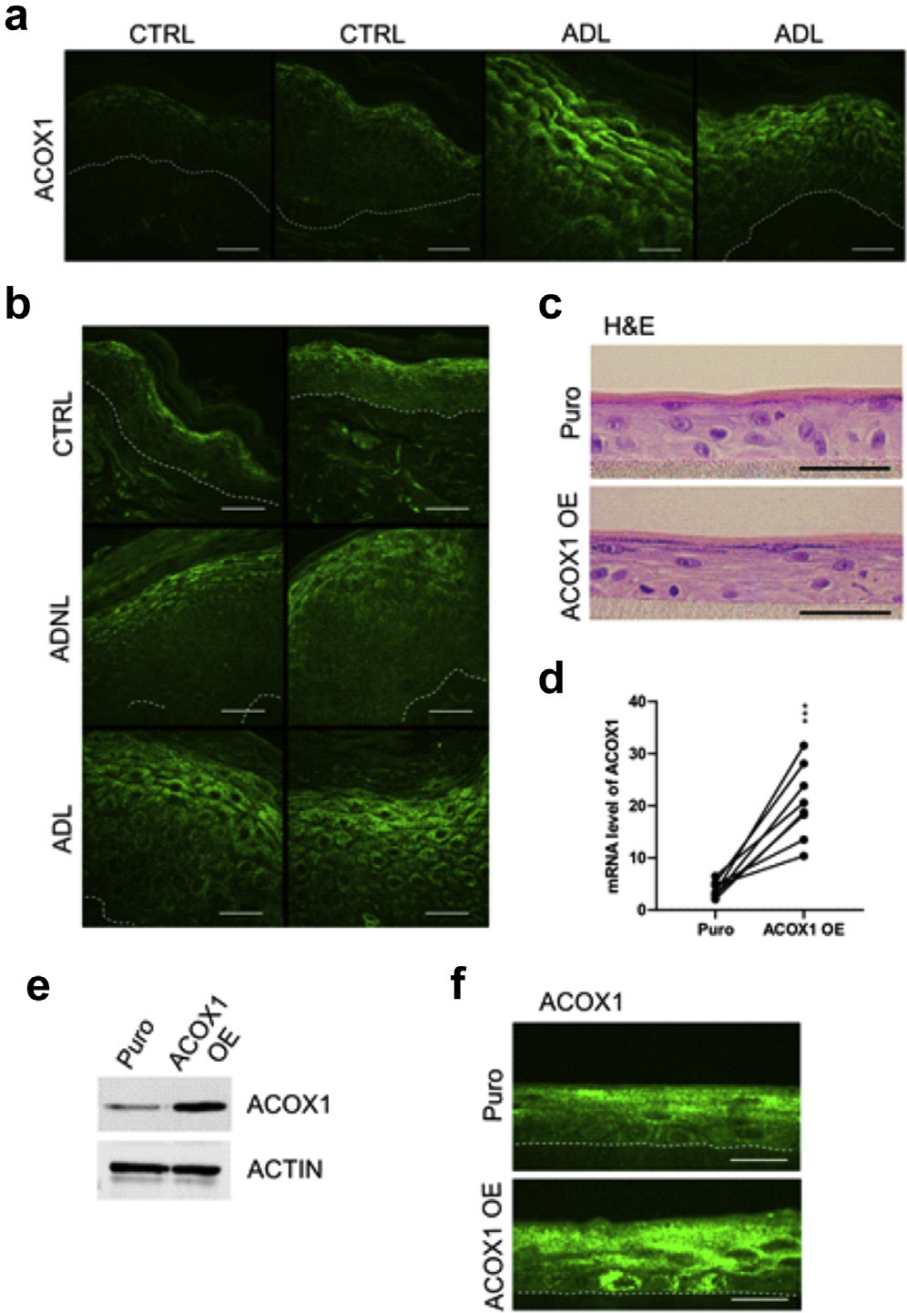
**Detection of ACOX1 in human AD skin and overexpression of ACOX1 in HEEs.** (**a, b**) Representative immunostaining showing the protein abundance of ACOX1 in ADL) and in ADNL epidermis compared with the epidermis of healthy donors (CTRL). The dashed line indicates the dermal‒epidermal boundary. Bar = 30 μm. (**c**) Representative H&E staining of HEEs generated with KCs infected with lentivirus containing either pHR-SFFV-ACOX1 (ACOX1 OE) or pHR-SFFV-Puro CTRL(Puro) vector. Bar = 50 μm. (**d**) mRNA and (**e, f**) protein levels of ACOX1 in HEEs overexpressing ACOX1 compared with those in their Puro CTRLs (n = 8). The dashed line indicates the basal epidermal layer. Bar = 50 μm. Data were analyzed with a paired Student’s *t*-test. ∗∗∗*P* < 0.001. AD, atopic dermatitis; ADL, lesional atopic dermatitis; ADNL, nonlesional atopic dermatitis; CTRL, control; HEE, human epidermal equivalent; KC, keratinocyte.

**Figure 5:**
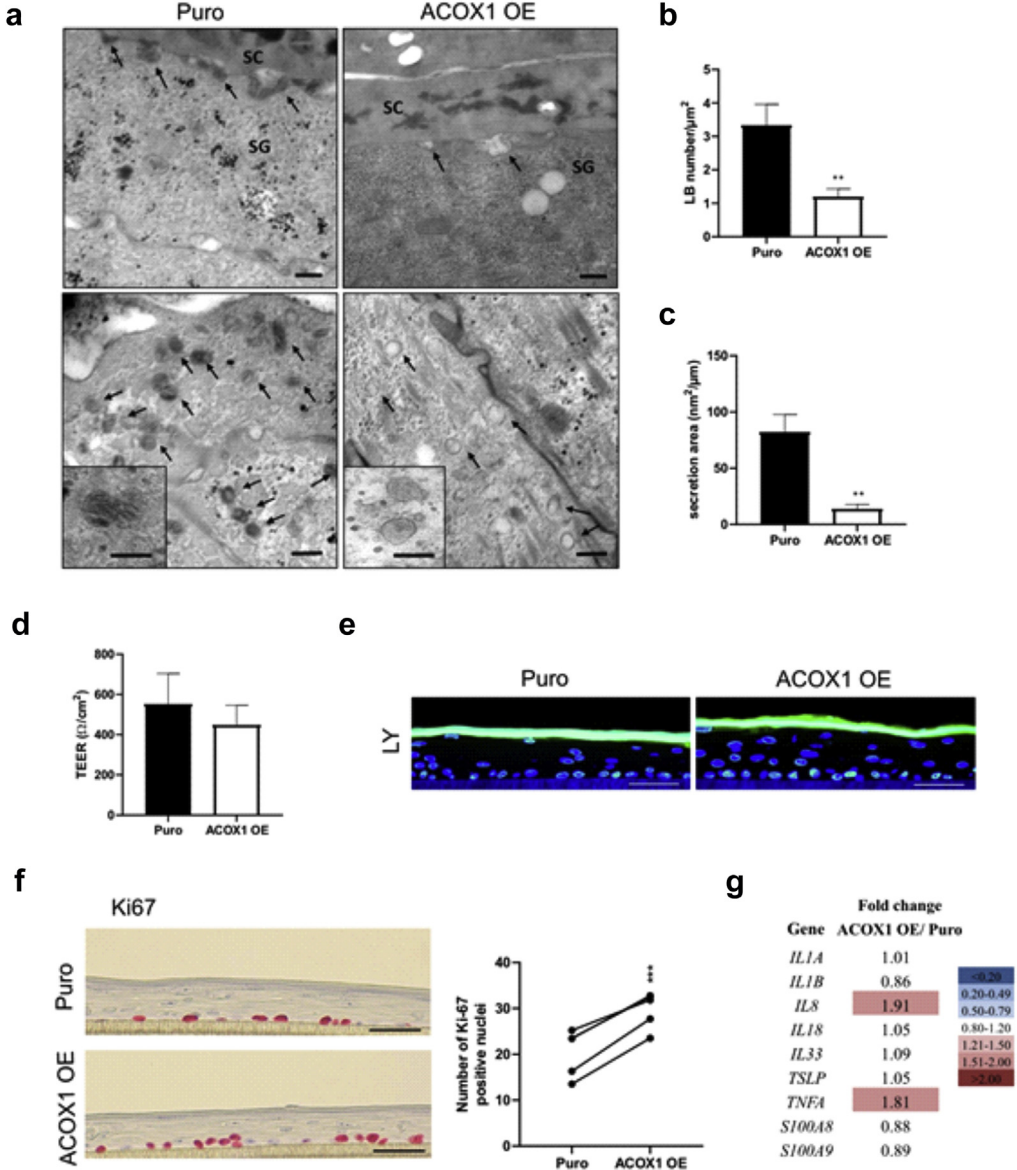
**Analysis of HEEs overexpressing ACOX1.** (**a**) Ultrastructural analysis showing LB secretion (upper panel, arrows), LB numbers (lower panel, arrows), and morphology (insets) in HEEs overexpressing ACOX1 (right panel) and in their Puro controls (left panel). Osmium tetroxide after fixation. Bar = 250 nm or 125 nm (inset). (**b**) LB numbers and (**c**) quantified secretion areas in HEEs in eight randomly selected fields per group (n = 2). (**d**) TEER and (**e**) LY penetration assay (green) in HEEs. Nuclei were counterstained with DAPI (blue). Bar = 50 μm. (n = 3). (**f**) Representative Ki-67 staining and the number of Ki-67‒positive nuclei in HEEs overexpressing ACOX1 compared with those in their Puro controls (n = 5). (**g**) Heat map showing the fold changes in the mRNA level of inflammation-related genes in HEEs (n = 5–7). Data were analyzed with a paired Student’s *t*-test. ∗∗*P* < 0.01 and ∗∗∗*P* < 0.001. HEE, human epidermal equivalent; LB, lamellar body; LY, Lucifer yellow; SC, stratum corneum; SG, stratum granulosum; TEER, transepithelial electrical resistance.

**Figure 6:**
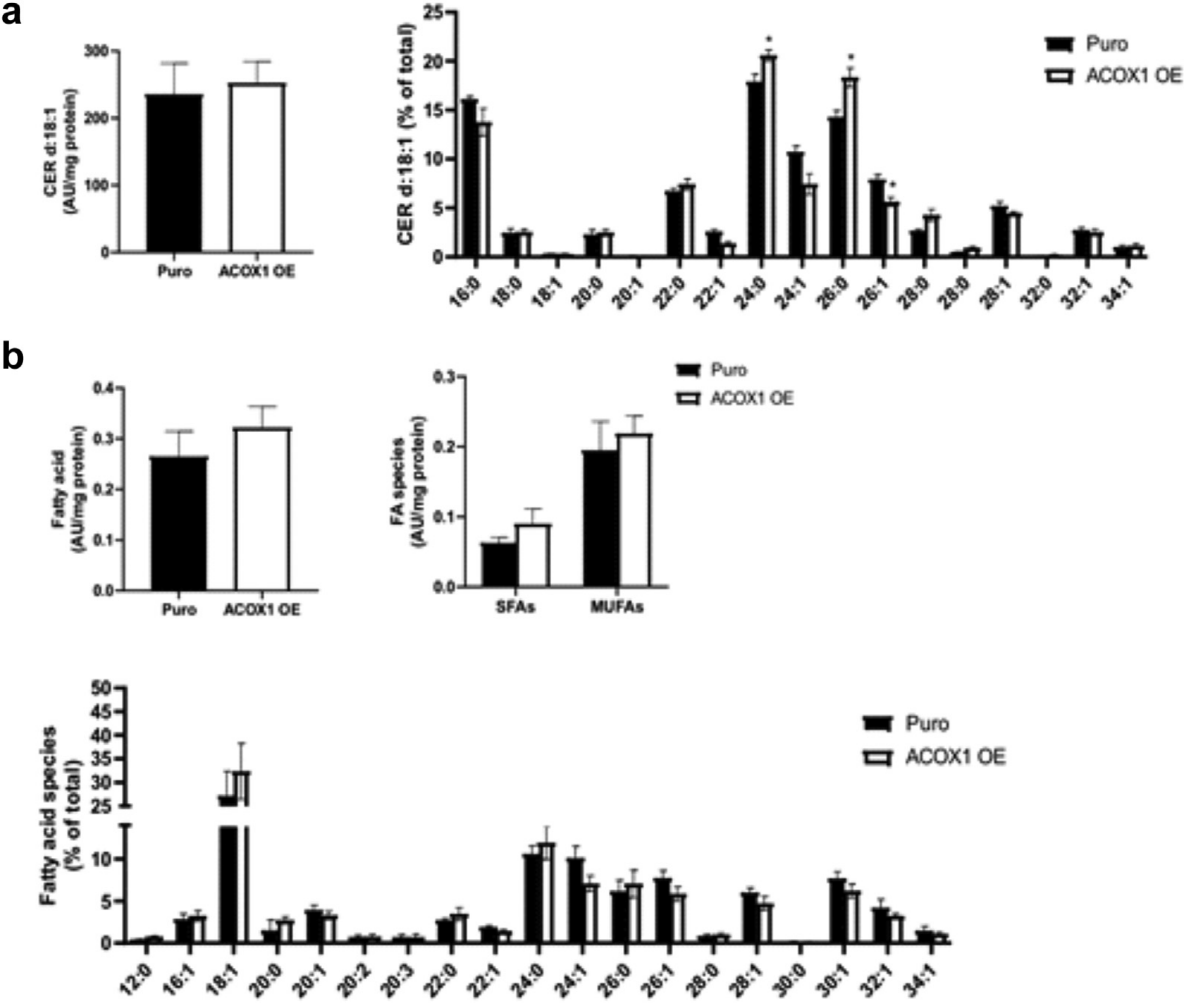
**Lipidomic analysis of HEEs overexpressing ACOX1.** (**a**) d18:1 Cers(NS) (n = 3) and (**b**) FFA (n = 3) species in HEEs generated with KCs infected with lentivirus containing either pHR-SFFV-ACOX1 (ACOX1 OE) or pHR-SFFV-Puro control (Puro) vector. Data are shown as analyte/IS ratio (AU) per mg protein or as the relative percentage of total lipid species. Data were analyzed with a Student’s *t*-test. ∗*P*< 0.05. AU: arbitrary unit; Cer, ceramide; FFA, free fatty acid; HEE, human epidermal equivalent; IS, internal standard; KC, keratinocyte; MUFA, monounsaturated fatty acid; SFA, saturated fatty acid.

To study the relevance of our findings in humans, we carried out immunostaining for ACOX1 in the skin of patients with AD and in those of healthy donors. We found a marked increase of ACOX1 in the spinous and granular layers of ADL epidermis compared with those in the healthy skin (Figure 4a), in contrast to nonlesional AD skin (Figure 4b). To investigate how increased ACOX1 in the human epidermis might contribute to ADL, we overexpressed ACOX1 in human KCs. Cells were infected with lentivirus particles encoding cDNA for human ACOX1 (pHR-SFFV-ACOX1) or puromycine resistance (pHR-SFFV-Puro [control]) before generating human epidermal equivalents (HEEs) (Figure 4c). Overexpression of ACOX1 was demonstrated by qPCR, Western blot analysis, and immunofluorescence staining (Figure 4d–f). Transmission electron microscopy revealed an overall decrease in the number of LBs in HEEs overexpressing ACOX1 compared with that in the Puro controls (Figure 5a and b). Moreover, ACOX1 overexpression was associated with reduced LB secretion and altered LB content, with many LBs containing inhomogeneous material or appearing as empty vesicles, in contrast to the Puro controls (Figure 5a–c). However, transepithelial electrical resistance did not differ, and Lucifer yellow did not penetrate the living layers of HEEs in both experimental groups (Figure 5d and e). This is in line with previous work showing no Lucifer yellow penetration in AD HEEs despite ultrastructural abnormalities of the SC and of the LB secretion system (Blunder et al., 2017; Leman et al., 2019). Furthermore, Ki67 staining showed that ACOX1 overexpression resulted in enhanced KC proliferation, but no noticeable histological differences were observed (Figure 5f). We screened the expression of various inflammatory mediators (i.e., *IL1A*, *IL1B*, *IL8*, *IL18*, *IL33*, *TSLP*, *TNFA*, *S100A8*, *S100A9*) and found a 1.9-fold increase in *IL8* mRNA level and a 1.8-fold increase in *TNFA* mRNA level, although these differences did not reach significance (Figure 5g). We did not observe differences in the expression of inflammatory cytokines between nontransfected and Puro control HEEs (data not shown). Furthermore, lipidomic analysis showed that relative levels of Cer(NS) containing C_24:0_ and C_26:0_ FA moiety were increased, whereas those containing C_24:1_ (nervonic acid) and C_26:1_ (hexacosenoic acid) were reduced in HEEs overexpressing ACOX1 compared with those in the Puro controls *(*Figure 6a), similar to FFA profile (Figure 6b). Nervonic and hexacosenoic acids are exclusively oxidized in peroxisomes and contribute to epidermal barrier formation (Cameron et al., 2007). Overall, these results show that ACOX1 overexpression enhances KC proliferation, potentially through *IL8* and *TNFA* upregulation (Steude et al., 2002; Tuschil et al., 1992), and concomitantly inhibits the LB secretion system.

### Synthesis and elongation of FAs are triggered in *ft/ft* mouse epidermis

**Figure 7:**
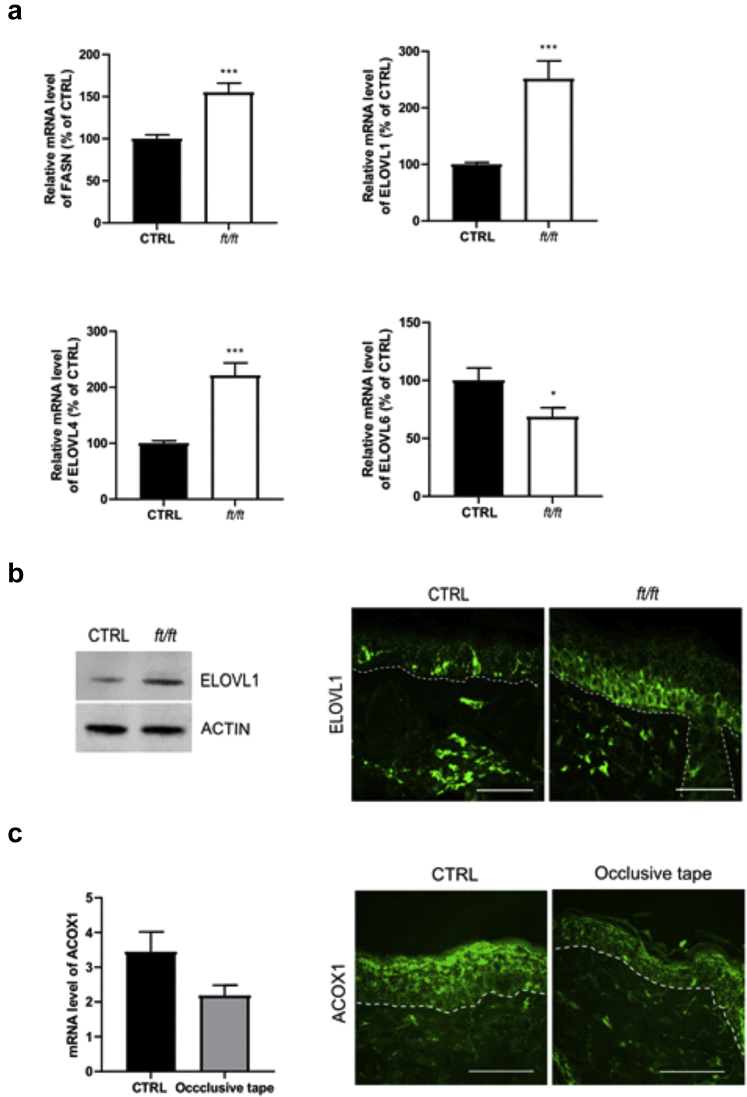
**Fatty acid synthesis and elongation in *ft/ft* mouse epidermis.** (**a**) Relative mRNA levels of *Fasn*, *Elov11*, *Elov4*, and *Elov6* in the epidermis of *ft/ft* mice compared with those in the epidermis of the CTRL mice (n = 9–10). (**b**) Representative western blot and immunostaining showing protein abundance of ELOVL1 in the epidermis of CTRL and *ft/ft* mice. (**c**) qPCR showing the relative mRNA level of *Acox1* (left panel) and its protein abundance (right panel) in the epidermis of *ft/ft* mice uncovered (CTRL) or covered with an occlusive dressing to reduce TEWL (n = 5). The dashed line indicates the dermal‒epidermal boundary. Bar = 50 μm. Data were analyzed with a Student’s *t*-test. ∗*P* < 0.05 and ∗∗∗*P* < 0.001. CTRL, control; TEWL, transepidermal water loss.

Recent research suggested that altered lipid biosynthesis as well as hampered FA elongation upstream of Cer synthesis may underlie the abnormal lipid profile of AD skin (Danso et al., 2017). Therefore, we measured the mRNA levels of genes encoding enzymes involved in FA synthesis and elongation and found that expressions of *Fasn*, *Elovl1,* and *Elovl4* were increased and that of *Elovl6* was decreased in the epidermis of *ft/ft* mice compared with those in the epidermis of the controls (Figure 7a). Accordingly, the protein level of ELOVL1 was increased in *ft/ft* mouse epidermis (Figure 7b). Thus, these results show that FA synthesis and elongation are triggered in *ft/ft* mouse epidermis. Because ELOVL1 and ELOVL4 elongate FAs from C_18_ up to C_38_, which are preferential substrates for peroxisomal oxidation (Lodhi and Semenkovich, 2014; Reddy and Hashimoto, 2001; Singh et al., 1984), it is likely that VLCFA synthesis and peroxisomal β-oxidation are coupled in *ft/ft* mouse epidermis. Because epidermal FFA composition exhibits a shift toward shorter FAs (Figure 1f), enhanced VLCFA synthesis in *ft/ft* mouse epidermis might not be able to compensate for their forced peroxisomal oxidation. Increased TEWL (Figure 1a) provokes heat loss (Bissinger and Annibale, 2010; Mathias et al., 1981; Wilson et al., 1988), and interestingly, peroxisomal β-oxidation of FAs per se generates heat and is involved in adaptive thermogenesis independent of UCP1 (Kramar, 1986; Lodhi and Semenkovich, 2014). To investigate the relationship between TEWL and ACOX1, we applied an occlusive dressing onto *ft/ft* mouse skin to reduce TEWL (Grubauer et al., 1989; Proksch et al., 1990). A reduction of ACOX1 at protein and mRNA levels (borderline nonsignificant) was observed in *ft/ft* mouse epidermis after skin occlusion (Figure 7c). Thus, in *ft/ft* mouse epidermis, VLCFA/ULCFA synthesis might be enhanced to provide peroxisomes with lipid substrates to fulfill bioenergetic requirements (e.g., KC proliferation/differentiation) and local thermogenesis owing to increased TEWL.

### Increased glycolysis in *ft/ft* mouse epidermis

**Figure 8:**
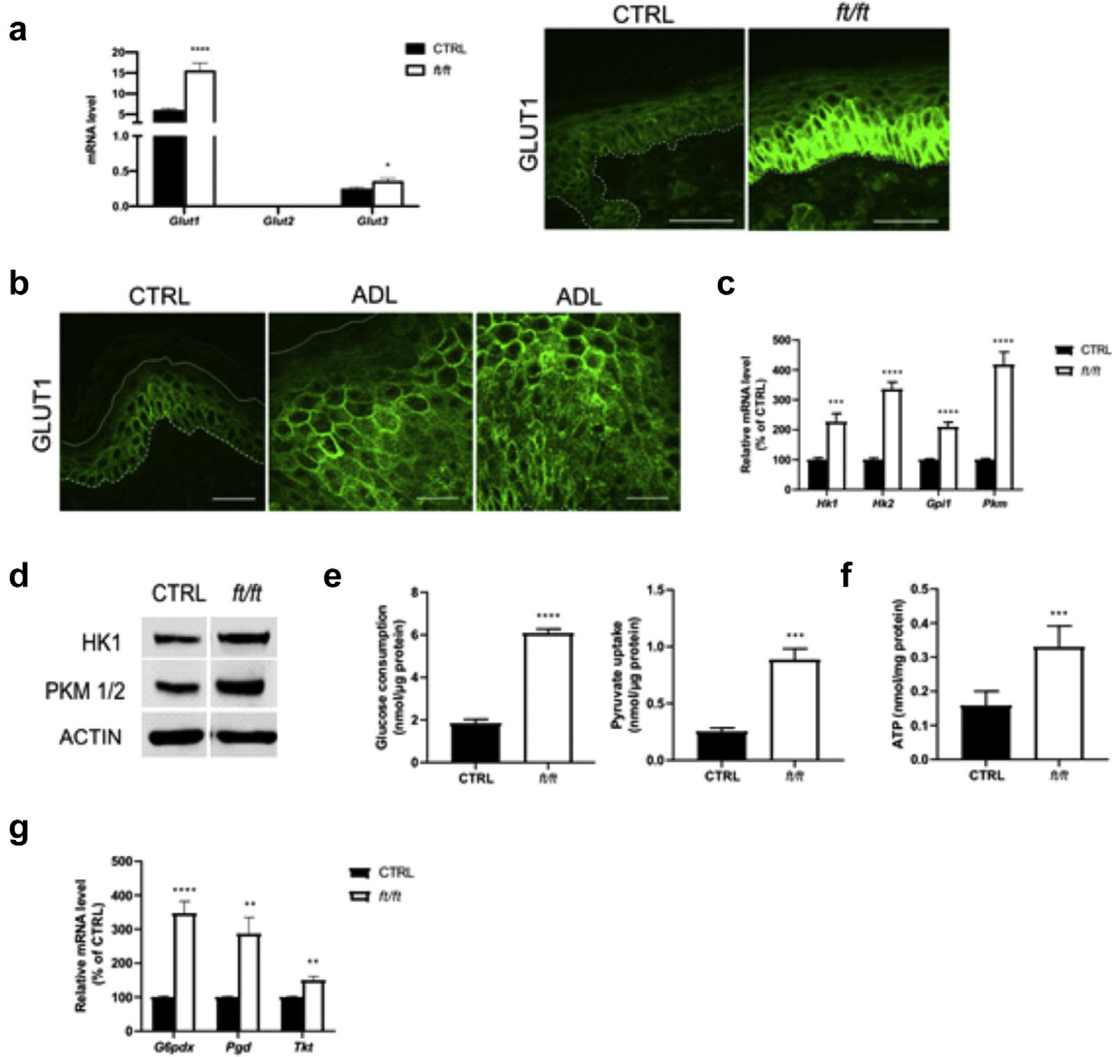
**Analysis of the glycolytic pathway in the epidermis of *ft/ft* mice.** (**a**) Relative mRNA levels of *Glut* isoforms and protein level of GLUT1 in the epidermis of CTRL and *ft/ft* mice (n = 9). Bar = 50 μm. (**b**) Immunostaining of GLUT1 in the epidermis of patients withADL and healthy donors (CTRL). The dashed line indicates the dermal‒epidermal boundary, and the continuous line denotes the SG‒SC interface. Bar = 30 μm. (**c, d**) Relative mRNA and protein levels of key glycolytic enzymes in mouse epidermis (n = 9–10). (**e**) Glucose and pyruvate consumption. (**f**) ATP level in the epidermis of CTRL and *ft/ft* mice (n = 5). (**g**) Relative mRNA level of key enzymes of the pentose phosphate pathway in mouse epidermis (n = 9–10). Data were analyzed with a Student’s *t*-test. ∗*P* < 0.05, ∗∗*P* < 0.01, ∗∗∗*P* < 0.001, and ∗∗∗∗*P* < 0.0001. AD, atopic dermatitis; ADL, lesional atopic dermatitis; ATP, adenosine triphosphate; CTRL, control; SC, stratum corneum; SG, stratum granulosum.

**Figure 9:**
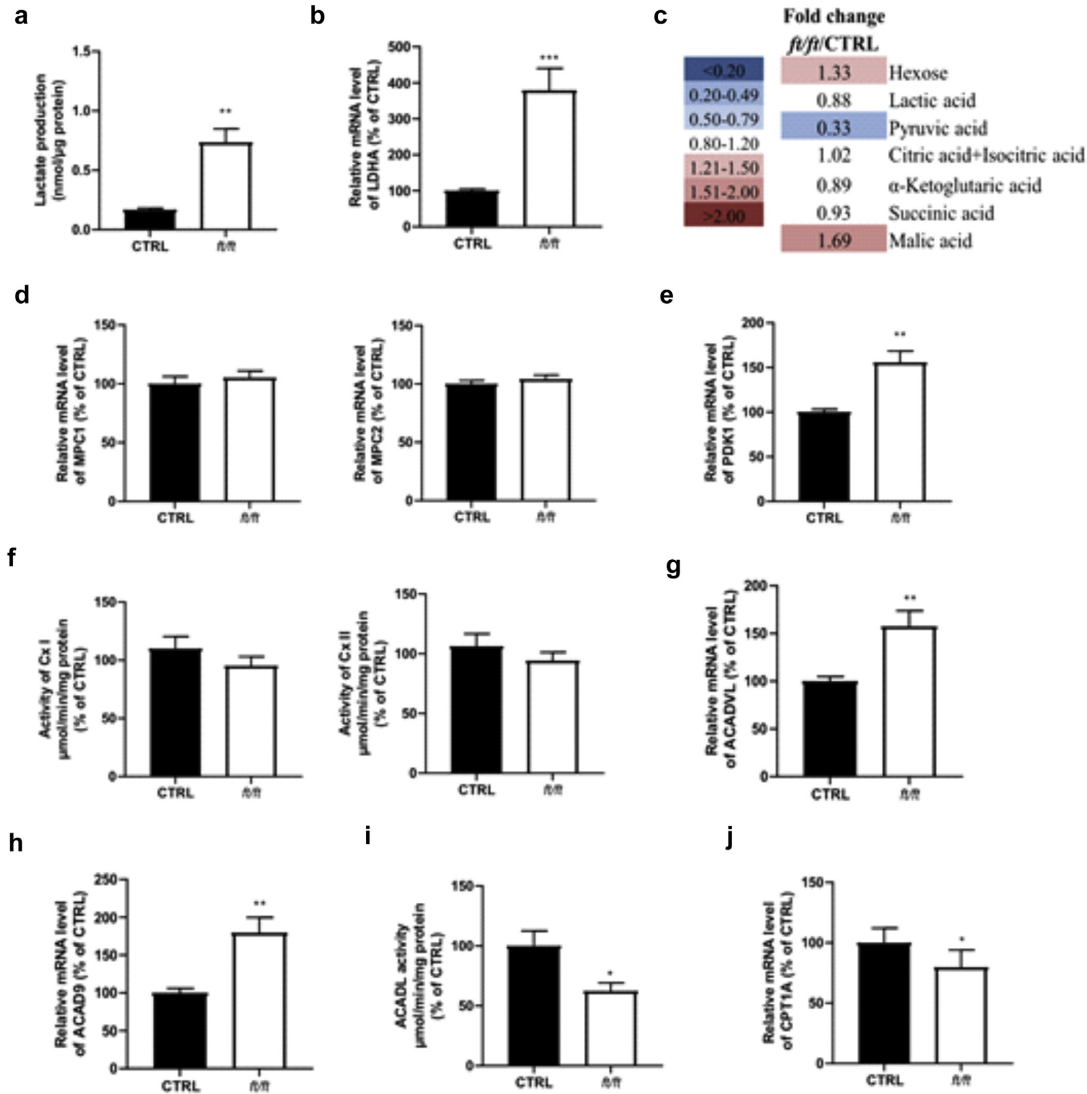
**Anaerobic glycolysis but not mitochondrial function is triggered in *ft/ft* mouse epidermis.** (**a**) Lactate production and (**b**) relative mRNA level of *Ldha* in mouse epidermis (n = 5–9). (**c**) Intracellular levels of the intermediates of the Krebs cycle in mouse epidermis quantified by LC‒MS. Fold changes between the mean values of five mice per group are shown. The list of all quantified metabolites as well as respective fold changes are provided in Table 4. (**d**) Relative mRNA expression of *Mpc1* and *Mpc2* in the epidermis of CTRL and *ft/ft* mice (n = 5–9). (**e**) Relative mRNA level of *Pdk1* in mouse epidermis (n = 9–10). (**f**) Activity of Cx I and of Cx II of the respiratory chain in mouse epidermal cells (n = 9–10). Relative mRNA level of (**g**) *Acadvl* and (**h**) *Acad9* in the epidermis of mice (n = 9–10). (**i**) Activity of mitochondrial ACADL measured in epidermal cells of CTRL and *ft/ft* mice (n = 10). (**j**) Relative mRNA level of *Cpt1a* in mouse epidermis (n = 5–9). Data were analyzed with a Student’s *t*-test. ∗*P* < 0.05, ∗∗*P* < 0.01, and ∗∗∗*P* < 0.001. CTRL, control; Cx, complex; LC‒MS, liquid chromatography‒mass spectrometry; min, minute.

**Figure 10:**
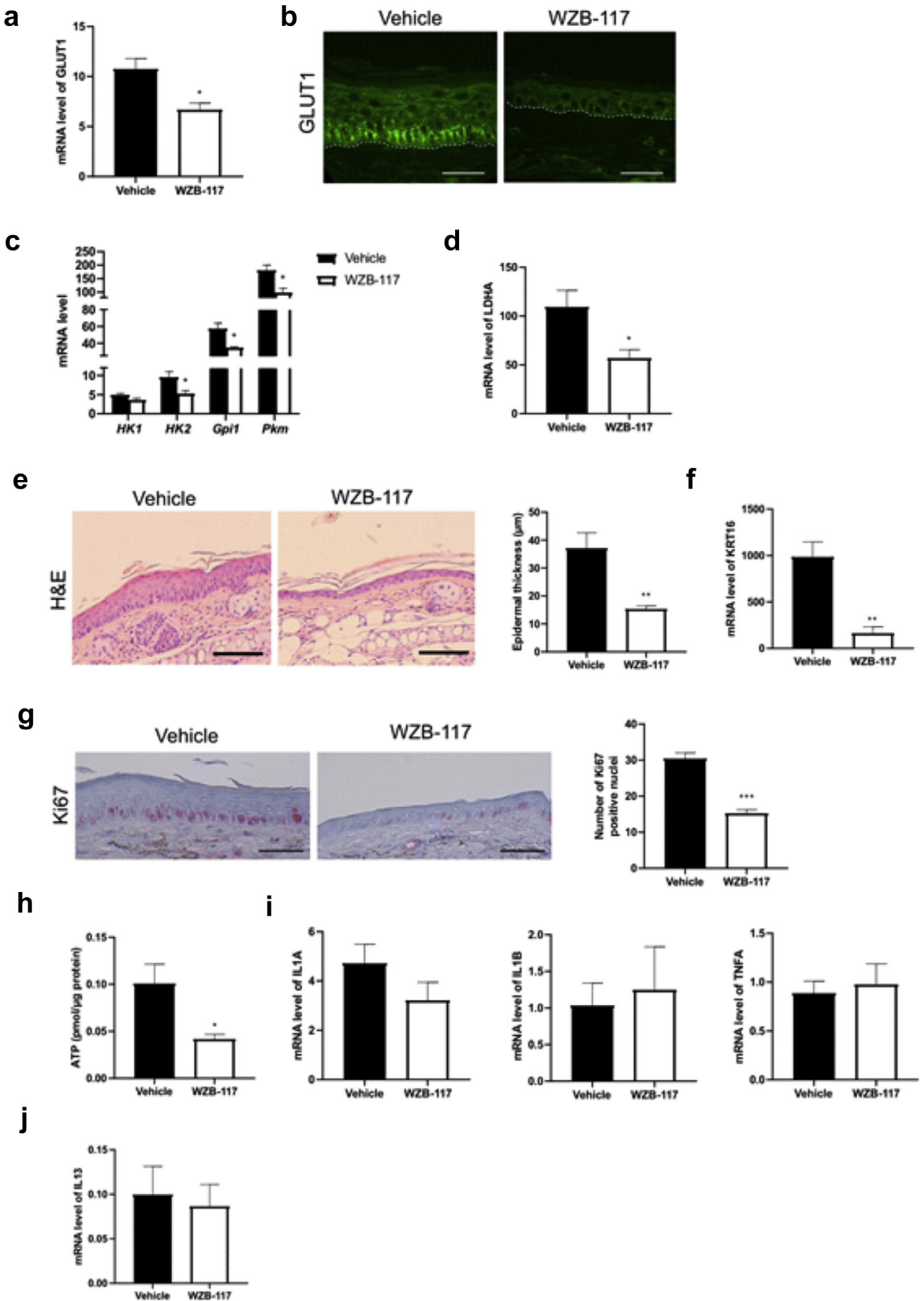
**Inhibition of glucose uptake in *ft/ft* mouse epidermis.** (**a**) mRNA and (**b**) protein levels of GLUT1 in the epidermis of mice treated with WZB-117 or vehicle CTRL (n = 4). (**c**) mRNA levels of key enzymes of glycolysis and of (**d**) *Ldha* in the epidermis of mice treated with WZV-117 compared with that in vehicle-treated CTRLs (n = 4). (**e**) Representative H&E staining of ear sections (Bar = 100 μm) and measured epidermal thickness and (**f**) mRNA level of keratin 16, that is, *K16*, in the epidermis of *ft/ft* mice treated with WZB-117 or vehicle CTRL (n = 4). (**g**) Representative Ki-67 staining and number of Ki-67^+^ nuclei in the epidermis of WZB-117 and vehicle-treated mice. Bar = 50 μm. (**h**) Intracellular ATP levels and (**i**, **j**) mRNA levels of inflammatory cytokines in the epidermis of mice treated with WZB-117 or vehicle CTRL (n = 4). Data were analyzed with a Student’s *t*-test. ∗*P* < 0.05, ∗∗*P* < 0.01, and ∗∗∗*P* < 0.001. ATP, adenosine triphosphate; CTRL, control; K, keratin.

We next questioned whether glucose might serve as an energy source to sustain KC proliferation and contribute to hyperplasia in ADL epidermis, similar to what has been evidenced in psoriasis (Zhang et al., 2018). We assessed the expression of glucose transporters in mouse epidermis and found that mRNA levels of *Glut1* and *Glut3* were significantly increased in *ft/ft* mouse epidermis compared with those in the control epidermis, although *Glut1* was the most abundant isoform (Figure 8a). Moreover, immunofluorescence staining showed a marked increase of GLUT1 in the basal layer and immediate spinous layer (Gherzi et al., 1992; Zhang et al., 2018) of *ft/ft* mouse epidermis compared with those of the control epidermis (Figure 8a), similar to that observed in those of the human ADL epidermis (Figure 8b). Moreover, the mRNA and protein levels of key glycolytic enzymes (HK1/2, GPI1*,* and, PKM) (Figure 8c and d) (Li et al., 2015) as well as glucose and pyruvate uptake (Figure 8e) and adenosine triphosphate (ATP) content (Figure 8f) were all significantly increased in *ft/ft* mouse epidermis compared with those in the control epidermis. mRNA levels of key proteins of the pentose phosphate pathway (*G6pdx*, *Pgd*, and *Tkt*)—a metabolic pathway parallel to glycolysis (Stincone et al., 2015)—were increased in the epidermis of *ft/ft* mice compared with those in the epidermis of the controls (Figure 8g). Mammalian cells generate ATP from glucose through glycolysis in the cytosol and oxidative metabolism through oxidative phosphorylation in the mitochondria. We found that lactate production (Figure 9a) and the mRNA level of *Ldha* were higher in the epidermis of *ft/ft* mice than in that of the controls (Figure 9b), suggesting a higher conversion of pyruvate into lactate. Yet, metabolomic profiling revealed increased intracellular concentrations of hexose but not of lactate or pyruvate in the epidermis of *ft/ft* mice (Figure 9c), suggesting a rapid excretion of lactate. The expression of mitochondrial pyruvate carrier subunits *Mpc1* and *Mpc2* remained unchanged (Figure 9d), suggesting no increased entry of pyruvate in the mitochondria in the epidermis of *ft/ft* mice. Moreover, the expression of pyruvate dehydrogenase kinase isoform 1, a key enzyme involved in metabolic switch toward predominant anaerobic glycolysis through the reduction of mitochondrial oxidative pathways (Warburg effect) (Liu and Yin, 2017), was increased in the epidermis of *ft/ft* mice in comparison with that of the controls (Figure 9e). In line with this, the concentrations of intermediates of the Krebs cycle were overall similar in the two groups (Figure 9c)—malic acid was increased. The Krebs cycle provides complexes I and II of the respiratory chain with reduced substrates, that is, NADH_2_ and FADH_2_, respectively. In line with unaltered Krebs cycle turnover, the activity of mitochondrial complexes I and II remained unchanged in the epidermis of *ft/ft* mice compared with that in the epidermis of the controls (Figure 9f). Our earlier results showed upregulated peroxisomal FA oxidation coupled with increased export of shortened FAs out of peroxisomes (Figure 2b–g) and increased expression of *Acadm*, *Acadl*, *Acadvl,* and *Acad9* (Figures 2a and 9g and h), altogether suggesting possible enhancement of mitochondrial oxidation of FAs (Lodhi and Semenkovich, 2014; Wanders et al., 2016). However, the enzymatic activity of mitochondrial ACADL was reduced in *ft/ft* mouse epidermis when compared with control epidermis (Figure 9i) as well as the expression of *Cpt1a* (Figures 2a and 9j). Thus, in *ft/ft* mouse epidermis, shortened FAs secreted out of the peroxisomes might be partly oxidized in mitochondria and might partly be used for synthesizing shorter Cers. Because these results show increased anaerobic glycolysis in *ft/ft* mouse epidermis, we next investigated the dependency of AD-like phenotype of *ft/ft* mice on this metabolic pathway. Ears of *ft/ft* mice were topically treated with WZB-117, a molecule blocking glucose uptake and glycolysis through GLUT1 inhibition (Liu et al., 2012; Zhang et al., 2018). Treatment with WZB-117 reduced mRNA and protein levels of GLUT1 (Figure 10a and b) and decreased mRNA levels of the key glycolytic genes *Hk2*, *Gpi1*, *Pkm,* and *Ldha* in the epidermis of *ft/ft* mice (Figure 10c and d). Moreover, topical treatment of *ft/ft* mice with WZB-177 significantly reduced epidermal hyperplasia as well as the mRNA level of Keratin 16, that is, *K16*) (Figure 10e and f) and the number of Ki67^+^ KCs (Figure 10g). Treatment of *ft/ft* mice with WZB-177 led to a drop in epidermal intracellular ATP content (Figure 10h) but did not reduce the epidermal expression of markers of barrier impairment such as *Il1a*, *Il1b,* and *Tnfa* or the expression of the T helper type-2 cytokine *Il13* (Figure 10i and j). Collectively, these results suggest that in *ft/ft* mouse epidermis, glycolysis and the pentose phosphate pathways—likely not mitochondria—provide KCs with ATP and macromolecules to fulfill high bioenergetic needs to sustain a high rate of proliferation. However, enhanced glycolysis does not contribute to local inflammation.

### FLG deficiency per se is not sufficient to alter epidermal lipid composition or to cause changes in KC energy metabolism

**Figure 11:**
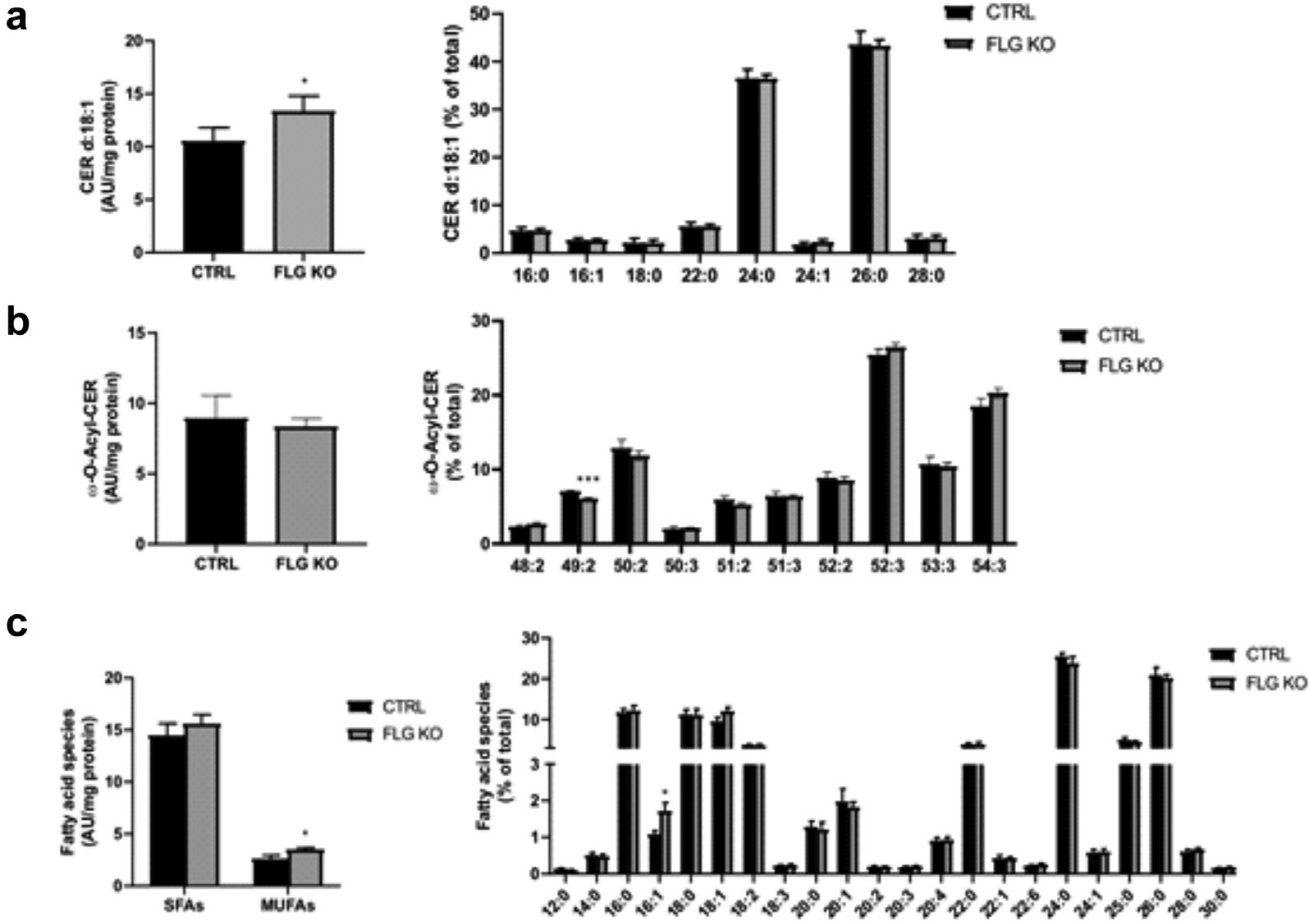
**Lipidomic analysis of *Flg*-KO mouse epidermis.** Relative and total amounts of (**a**) d18:1 Cers(NS), (**b**) ω-O-acylCers (n = 5), (**c**) SFAs, and MUFAs in the epidermis of *Flg*-KO mice compared with those in the epidermis of CTRL mice (n = 5). Data are shown as analyte/IS ratio (AU) per mg protein or as the relative percentage of total lipid species. Data were analyzed with a Student’s *t*-test. ∗*P* < 0.05 and ∗∗∗*P* < 0.001. AU, arbitrary unit; Cer, ceramide; CTRL, control; *Flg*-KO, *Flg*-knockout; IS, internal standard; KO, knockout; MUFA, monounsaturated fatty acid; SFA, saturated fatty acid.

**Figure 12:**
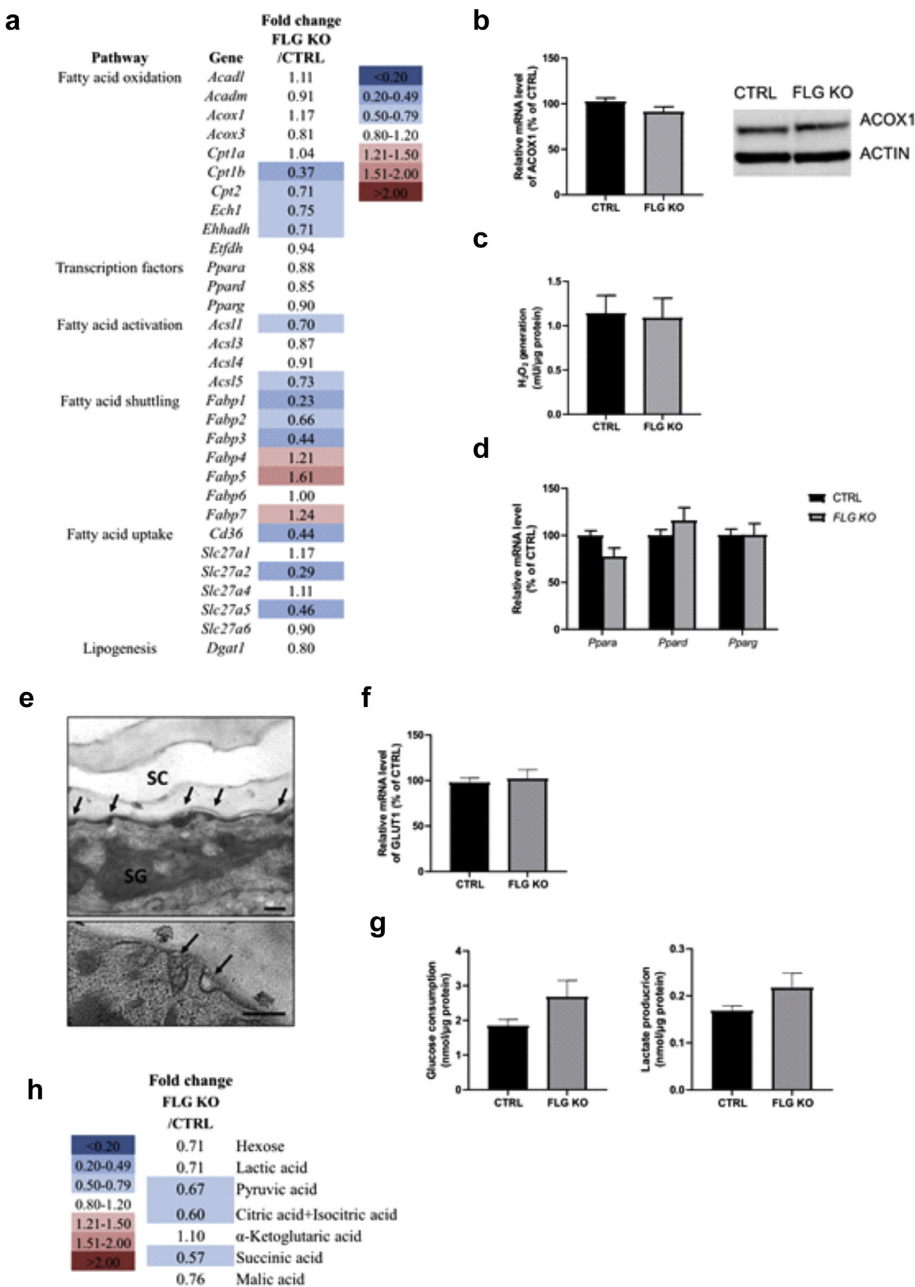
**Metabolic and ultrastructural analysis of *Flg*-KO mouse epidermis.** (**a**) Microarray analysis showing fold changes of a subset of genes involved in FA metabolism in the epidermis of *Flg*-KO mice compared with those in the epidermis of CTRLs. (**b**) Relative *Acox1* mRNA level (n = 10) and protein abundance as well as (**c**) ACOX activity in CTRL and *Flg*-KO mouse epidermis (n = 5). (**d**) Relative levels of PPAR mRNA, *Ppar*, isoforms in mouse epidermis (n = 10). (**e**) Ultrastructural analysis of *Flg*-KO mouse epidermis showing LB morphology and secretion (arrows). Osmium tetroxide after fixation. Bar = 250 nm. (**f**) Relative mRNA level of *Glut1* in mouse epidermis (n = 5). (**g**) Glucose consumption and (**d**) lactate production by epidermal sheets of CTRL and *Flg*-KO mice (n = 5). (**h**) Intracellular levels of metabolites and Krebs cycle intermediates quantified by LC‒MS in the epidermis of *Flg*-KO mice compared with those in the epidermis of CTRLs. Fold changes between the mean values of five mice per group are shown (n = 5). Data were analyzed with a Student’s *t*-test. The list of genes featured on the array and of all quantified metabolites as well as respective fold changes are provided in Tables 3 and 4. CTRL, control; *Flg*-KO, *Flg*-knockout; KO, knockout; LB, lamellar body; LC‒MS, liquid chromatography‒mass spectrometry; PPAR, peroxisome proliferator–activated receptor; SC, stratum corneum; SG, stratum granulosum.

Because *ft/ft* mice carry two gene mutations, that is, *Flg* and *Tmem79* (Sasaki et al., 2013), we next sought to determine the contribution of FLG deficiency to abnormalities in lipid metabolism using *Flg*-knockout (KO) mice (Kawasaki et al., 2012; Saunders et al., 2013). Total amounts of Cer(NS) and monounsaturated FAs were increased in the epidermis of *Flg*-KO mice when compared with that of the controls in absence of changes in chain length distribution of epidermal lipids (Figure 11a–c). Moreover, the mRNA levels of all *Ppar* isoforms as well as the mRNA level, protein abundance, and activity of ACOX1 were unaltered in the *Flg*-KO mouse epidermis compared with those in the control epidermis (Figure 12a–d). The expression of most lipid metabolic genes was reduced, with the exception of *Fabp* expression, which was moderately increased (Figure 12a). Despite normal lipid composition, transmission electron microscopy showed a heterogeneous population of LBs exhibiting both normal and abnormal content and secretion in *Flg*-KO mouse epidermis, albeit less pronounced than in the epidermis of *ft/ft* mice (Figure 12e). Furthermore, the mRNA level of *Glut1* was unaltered, and no changes in glucose uptake or lactate production were observed in the *Flg*-KO mouse epidermis compared with those in the control epidermis (Figure 12f and g). Metabolomic profiling of the epidermis revealed low intracellular concentrations of pyruvate but unchanged levels of hexose and lactate in *Flg*-KO mice compared with those in the controls (Figure 12h). Moreover, concentrations of the intermediates of the Krebs cycle were reduced or unchanged in the *Flg*-KO epidermis compared with those in the control epidermis (Figure 12h). Thus, FLG deficiency per se does not alter lipid or glucose metabolism in KCs, and the abnormalities in LB cargo composition observed in FLG-deficient mice, similar to what has been observed in patients with various ichthyoses, might rather result from cytoskeletal abnormalities, as suggested earlier (Elias and Wakefield, 2014; Gruber et al., 2011; Reynier et al., 2019). Moreover, our results are in line with those of previous work showing that the lipid phenotype of atopic skin is independent of *FLG* status (Janssens et al., 2012; van Smeden et al., 2014b).

### Enhanced peroxisomal β-oxidation and anaerobic glycolysis are independent of *Tmem79* mutation

**Figure 13:**
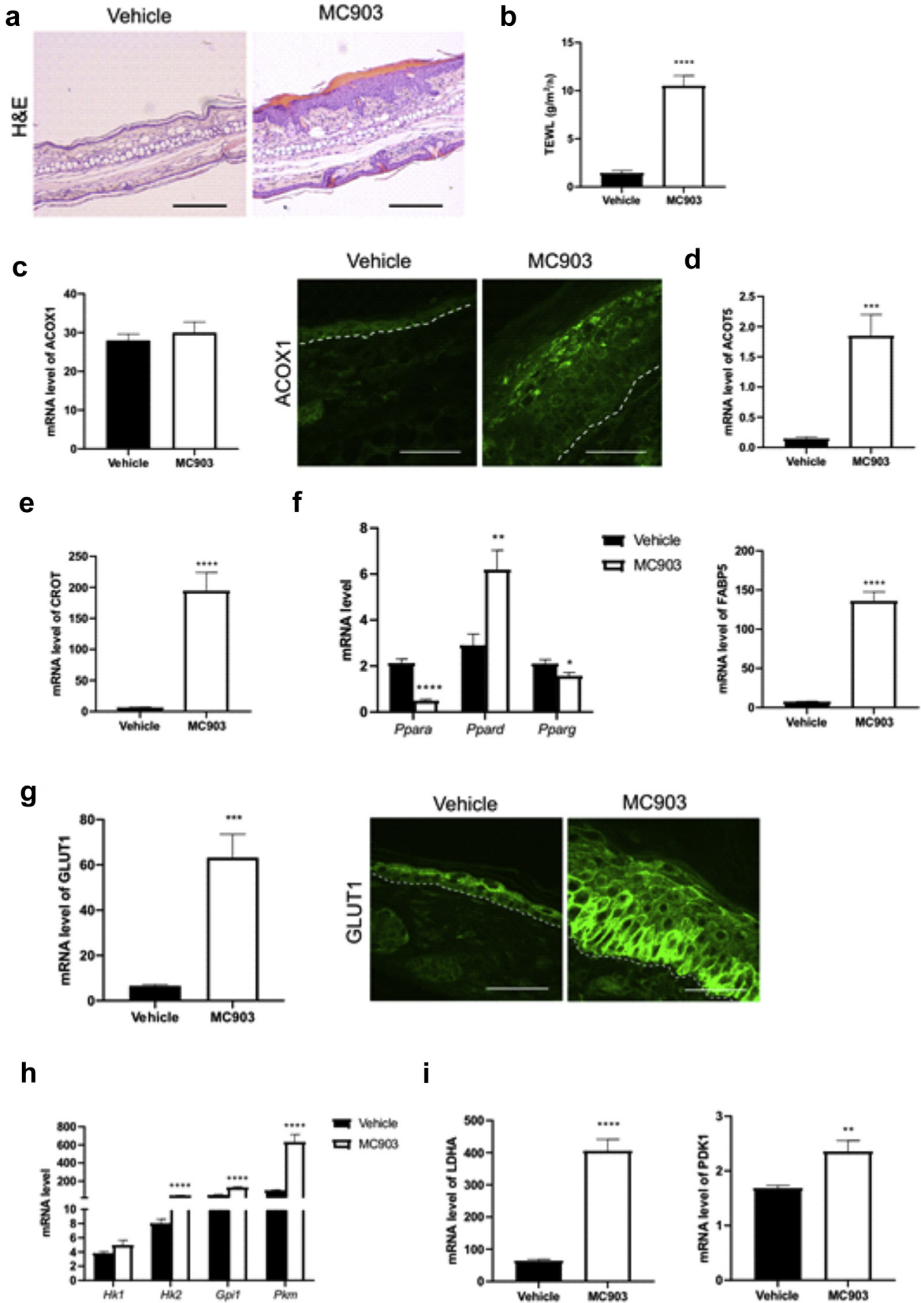
**MC903 mouse model of ADL** (**a**) Representative H&E staining of ear sections (bar = 200 μm) of MC903-treated mice compared with that of the vehicle-treated CTRLS (n = 7) and (**b**) TEWL values measured on the ears of mice (n = 7). (**c**) qPCR and immunostaining showing mRNA and protein level of ACOX1 in the epidermis of mice treated with MC903 or vehicle. The dashed line indicates the dermal‒epidermal boundary. Bar = 50 μm. mRNA level of (**d**) *Acot5*; (**e**) *Crot*; (**f**) PPAR mRNA, *Ppar*, isoforms; and *Fabp5* in the epidermis of mice treated with MC903 or vehicle (n = 7). (**g**) mRNA level and protein abundance of GLUT1 and (**h**) mRNA level of key enzymes of glycolysis as well as of (**i**) *Ldha* and *Pdk1* in the epidermis of mice treated with MC903 or vehicle (n = 7). The dashed line indicates the dermal‒epidermal boundary. Bar = 50 μm. Data were analyzed with a Student’s *t*-test. ∗*P* < 0.05, ∗∗*P* < 0.01, ∗∗∗*P* < 0.001, and ∗∗∗∗*P* < 0.0001. ADL, lesional atopic dermatitis; CTRL, control; PPAR, peroxisome proliferator–activated receptor; TEWL, transepidermal water loss.

To investigate whether the observed metabolic changes were specific to *ft/ft* mice, we utilized another mouse model of ADL exhibiting increased TEWL, that is, mice topically treated with MC903 (Figure 13a and b) (Elentner et al., 2009; Li et al., 2006; Moosbrugger-Martinz et al., 2017). We found increased ACOX1 levels in the granular KCs of the epidermis of MC903-treated mice compared with those in the epidermis of vehicle-treated controls, despite an unchanged mRNA level (Figure 13c). Furthermore, MC903 treatment enhanced epidermal expression of *Acot5*, *Crot* (Figure 13d and e), *Ppard,* and *Fabp5,* as opposed to the expression of *Ppara* or *Pparg* (Figure 13f). With respect to glycolysis, MC903-treated mice displayed increased epidermal GLUT1 (Figure 13g) and expression of key glycolysis-related enzymes (*Hk2*, *Gpi1* and *Pkm, Ldha, Pdk1*) (Figure 13h and i). Thus, these results suggest that enhanced peroxisomal β-oxidation and anaerobic glycolysis are not specific to *ft/ft* mice and are independent of *Tmem79* mutation.

### ACOX1 is enhanced in psoriatic skin

**Figure 14:**
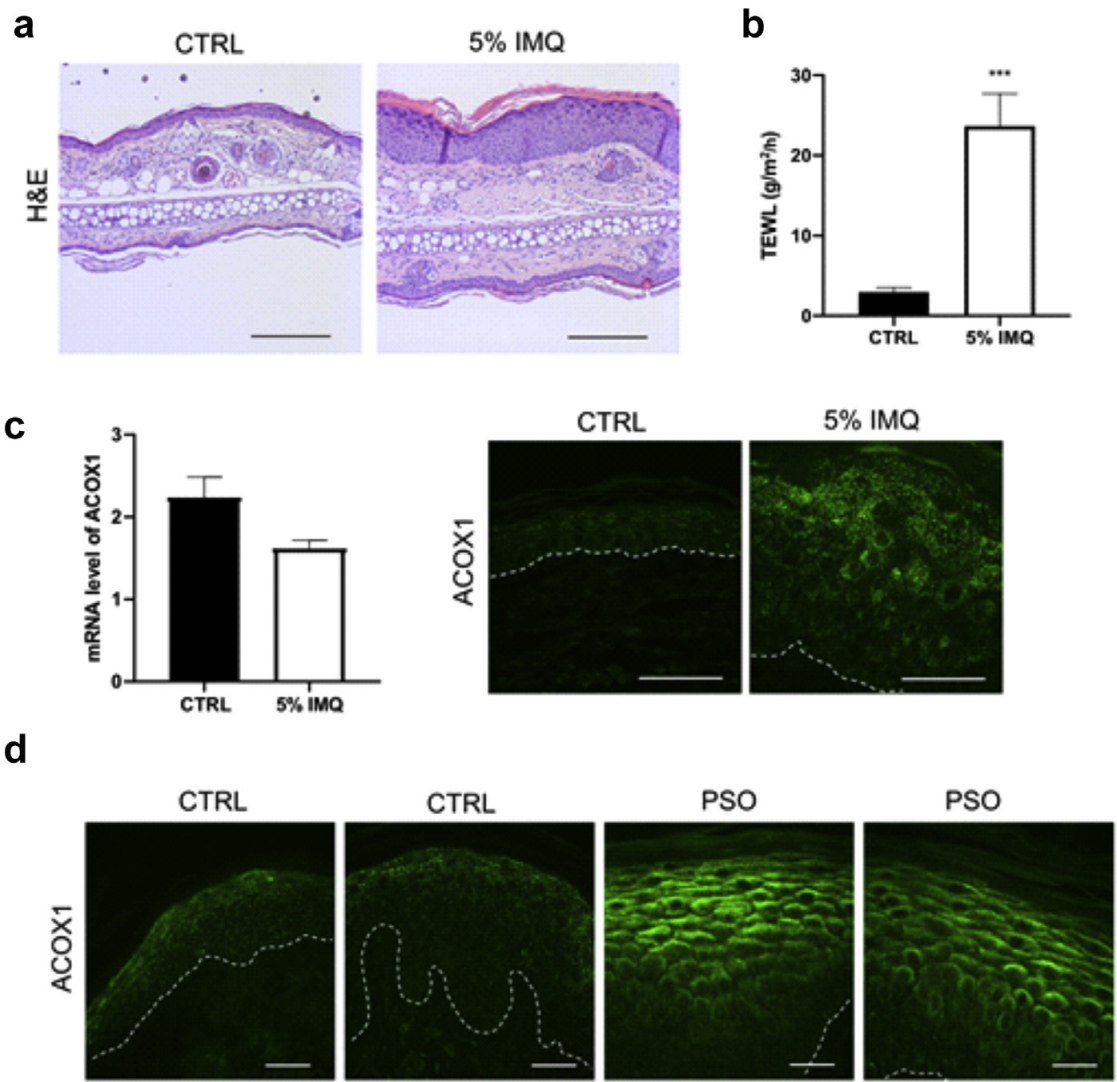
**IMQ mouse model and biopsies from patients with PSO.** (**a**) Representative H&E staining of ear sections (bar = 200 μm) of mice treated with 5% IMQ cream or with petroleum jelly (CTRL) (n = 7) and (**b**) TEWL values measured on the ears of mice (n = 7). (**c**) qPCR and immunostaining showing mRNA and protein level of ACOX1 in the epidermis of mice treated with 5% IMQ or with petroleum jelly. The dashed line indicates the dermal‒epidermal boundary. Bar = 50 μm. (**d**) Immunostaining of ACOX1 in the epidermis of patients with PSO and healthy donors (CTRL). The dashed line indicates the dermal‒epidermal boundary. Bar = 30 μm. Data were analyzed with a Student’s *t*-test. ∗∗∗*P* < 0.001. CTRL, control; IMQ, imiquimod; PSO, psoriasis; TEWL, transepidermal water loss.

Because ultra-long Cers such as C_26_ Cers are decreased in the epidermis of patients with psoriasis (Li et al., 2020; Łuczaj et al., 2020; Tawada et al., 2014), we wondered whether ACOX1 could be enhanced in psoriatic epidermis. Mice topically treated with imiquimod (IMQ) exhibited psoriasiform inflammation associated with increased TEWL (Figure 14a and b) as reported earlier (Horváth et al., 2019; Jabeen et al., 2020; van der Fits et al., 2009). Despite unchanged mRNA level (Figure 14c), immunostaining revealed enhanced ACOX1 in the upper epidermis of IMQ-treated mice compared with that in the upper epidermis of petroleum jelly-treated controls (Figure 14c), similar to that observed in human lesional psoriatic epidemis (Figure 14d). These results might confirm a tight relationship between TEWL and ACOX1.

## Discussion

Changes in metabolic states have been described in psoriatic, UV-irradiated, and wounded skin; in skin immune cells on activation; and in skin cancers (Hosseini et al., 2018, 2017; Pan et al., 2017; von Meyenn et al., 2019). However, whether AD causes adaptive metabolic changes in KCs has not been previously explored. This prompted us to study epidermal lipid and glucose metabolism in two mouse models of ADL, that is, *ft/ft* mice and mice topically treated with MC903. Results were compared with findings in psoriasis using a mouse model, that is, mice topically treated with IMQ. Moreover, skin biopsies from patients with ADL or psoriasis were utilized to validate important results in humans.

The causes of the impaired epidermal barrier in AD are complex (Cork et al., 2009; Leung, 2016; Leung and Guttman-Yassky, 2014). Yet, an important factor is likely the shortening of the lipid chain length in the SC (Czarnowicki et al., 2017; Ishikawa et al., 2010; Janssens et al., 2012; van Smeden et al., 2014b). Although the *ft/ft* mice are a well-accepted model of AD, exhibiting epidermal barrier defect as shown by increased TEWL (Figure 1a) (Moniaga et al., 2010; Sasaki et al., 2013; Saunders et al., 2013), the epidermal lipid composition of these mice had not been characterized previously. In this work, we revealed characteristic shifts toward shorter molecular species in all main lipid classes, that is, Cer(NS), ω-O-acylCer, and FFAs, in the epidermis of *ft/ft* mice (Figure 1b–f), mimicking the changes observed in human ADL (Ishikawa et al., 2010; Janssens et al., 2012; van Smeden et al., 2014b).

In the human SC, C_24:0_ and C_26:0_ FAs account for more than 50% of the total FFA content (van Smeden et al., 2014a), and their levels are reduced in AD, as are the levels of lipid species containing C_24_ and C_26_ FAs (Berdyshev et al., 2018; van Smeden et al., 2014b). Interestingly, C_24_ and C_26_ FAs are exclusively oxidized in peroxisomes (Lodhi and Semenkovich, 2014; Reddy and Hashimoto, 2001; Rizzo et al., 2003; Singh et al., 1984). ACOX1 catalyzes the first step of the oxidation of straight-chain VLCFAs and ULCFAs in peroxisomes (Lodhi and Semenkovich, 2014) and was increased in the epidermis of *ft/ft* (Figure 2b–d) or MC903-treated (Figure 13c) mice and in the epidermis of patients with ADL (Figure 4a and b). Moreover, by functional assay, we showed increased oxidation of lignoceroyl-CoA (C_24:0_-CoA) and hexacosanoyl-CoA (C_26:0_-CoA) in epidermal cells of *ft/ft* mice compared with that in epidermal cells of the controls (Figure 2e). Furthermore, in *ft/ft* mouse epidermis, we observed an increase in the expression of several genes involved in the synthesis of VLCFAs (Figure 7a), in contrast to genes involved in their uptake (Figure 2a). Therefore, we speculate that newly synthesized VLCFAs might be, in part, tethered to peroxisomes to be oxidized, instead of being utilized for barrier lipid assembly. In addition, lipid synthesis and uptake are likely not able to fully restore the pool of VLCFAs in KCs (Figure 1f). Thus, enhanced ACOX1-mediated FA oxidation might significantly contribute to the overall decline in VLCFAs/ULCFAs and to the shift toward shorter lipids, including Cers, in ADL epidermis. Interestingly, we observed empty LBs and disturbed LB exocytosis as well as reduced amounts of C_24:1_ and C_26:1_ and of Cers with C_24:1_ and C_26:1_ FA moiety in HEEs overexpressing ACOX1 (Figures 5a–c and 6a and b). Previous work showed lower amounts of ultra-long Cers (Li et al., 2020; Tawada et al., 2014) and of hexacosanoic acid in Cer(d18:0) in KCs of patients with psoriasis (Łuczaj et al., 2020), similar to what has been observed in KCs of patients with ADL (Berdyshev et al., 2018; van Smeden et al., 2014b). Moreover, abnormal LB secretion and lamellar bilayer have been evidenced in psoriasis (Ghadially et al., 1996). We found increased epidermal ACOX1 in both human psoriatic lesions and in mice treated with IMQ (Figure 14c and d). Thus, altogether, these results suggest a role of increased ACOX1 activity in abnormal LB secretion and epidermal lipid composition.

Excessive TEWL, as observed in ADL and involved psoriasis, is a manifestation of major impairment of the permeability barrier (Seidenari and Giusti, 1995; Yazdanparast et al., 2019). The application of an occlusive dressing onto *ft/ft* mouse skin to dampen TEWL resulted in the reduction of epidermal ACOX1 (Figure 7c). Enhanced peroxisomal β-oxidation through ACOX1 has been shown to be involved in the direct production of heat (Kramar, 1986; Lodhi and Semenkovich, 2014) and might compensate for superficial heat loss at skin sites with elevated TEWL (Bissinger and Annibale, 2010; Wilson et al., 1988). Thus, the upregulation of the ACOX1 pathway might primarily be a response to increased TEWL—not a response to high KC turnover—and be a double-edged compensatory mechanism, particularly consuming C_24_ and C_26_ lipids. It remains unclear how increased TEWL triggers ACOX1 upregulation. However, an enhanced PPARδ pathway induced by an impaired epidermal barrier (Blunder et al., 2021; Michalik et al., 2003; Tsai et al., 1994; Wood et al., 1992) might significantly contribute to ACOX1 upregulation (Higgins et al., 2012; Luquet et al., 2005) in both ADL- and psoriasis-like inflammation (Yamane et al., 2009).

Compensatory mechanisms marshaled in response to major epidermal barrier impairment include high KC turnover (Moniaga et al., 2010; Sasaki et al., 2013; Saunders et al., 2013; Suárez-Fariñas et al., 2011), which is an energy-demanding process that requires ATP and precursors for biosynthesis of macromolecules. Although less efficient than oxidative phosphorylation, anaerobic glycolysis generates ATP at a much faster rate and supplies cells with carbon molecules that could be shunted toward important anabolic pathways (e.g., biosynthesis of nucleotides, lipids, and proteins) (Bender and Martinou, 2016; de la Cruz-López et al., 2019; Liberti and Locasale, 2016; Urbańska and Orzechowski, 2019). We found upregulated *Glut1* mRNA and protein levels, increased glucose uptake, and increased expression of key glycolytic enzymes in the epidermis of *ft/ft* and MC903-treated mice compared with those in the epidermis of their respective controls (Figures 8a and c–e and 13g and h). Furthermore, functional assays and metabolite profiling revealed that anaerobic glycolysis rather than oxidative phosphorylation was triggered in *ft/ft* mouse epidermis (Figure 9). Moreover, topical treatment with a GLUT inhibitor significantly reduced epidermal hyperplasia and ATP content in *ft/ft* mice but did not ameliorate the local inflammation associated with either barrier impairment or the T helper type-2 immune response (Figure 10e–j). Thus, the high proliferation rate of KCs, as observed in ADL and psoriasis, likely triggers a metabolic shift toward anaerobic glycolysis (Zhang et al., 2018).

In summary, our results suggest that high TEWL, leading to heat loss, triggers epidermal peroxisomal β-oxidation of VLCFAs/ULCFAs potentially destined for the SC lipid matrix, which might ultimately contribute to the shift toward shorter lipid species, including Cers, as observed in ADL and psoriasis. High KC turnover in the epidermis increases anaerobic glycolysis in mouse models exhibiting ADL-like inflammation, similar to psoriasis.

## Materials and Methods

### Mice

*Ft/ft* mice were bred on a pure C57BL/6 background (kind gift from M. Amagai). Mice of the inbred strain C57BL/6 were purchased from Charles River Laboratories (Sulzfeld, Germany). *Flg*-KO mice (BRC number 05850: B6.Cg-Flg<tm1>) on a pure C57BL/6 background were purchased from RIKEN (Tsukuba, Japan). Age-matched female mice were used at age >24 weeks. Mouse care and use were approved by the Austrian Federal Ministry of Science and Research and were performed according to institutional guidelines (BMWFW-66.011/0199-WF/V/3b/2016).

### Processing of mouse tissue and KC isolation

Mouse epidermis was isolated by trypsinization (Sigma-Aldrich, St. Louis, MO) for 30 minutes at room temperature. For experiments using dorsal skin, mice were shaved, and their hair was removed using Veet hair removal cream 72 hours before sample collection. KCs were isolated and cultured as described earlier (Blunder et al., 2017).

### Treatment of mice

For inhibition of glucose transport, 1 mg/ml WZB-117 dissolved in acetone (Sigma-Aldrich) or vehicle (100% acetone) was applied topically once daily onto the ears of female *ft/ft* mice for 9 days according to previous work (Zhang et al., 2018). The epidermis was collected 6 hours after the last treatment. Psoriasis-like skin inflammation was induced in adult, age-matched, female C57BL/6 mice by topical application of a daily dose of 12.5 mg of Aldara cream (Meda Pharma GmbH, Frankfurt, Germany), containing 5% IMQ onto ears for 5 days (van der Fits et al., 2009). Control mice were topically treated with petroleum jelly. The epidermis was collected 24 hours after the last treatment. To induce ADL, 45 μM MC903 (Sigma Aldrich) in 12.5 μl ethanol was topically applied onto both sides of the ears of female C57BL/6 mice daily for 9 days consecutively (Moosbrugger-Martinz et al., 2017). Control mice were topically treated with ethanol (vehicle). The epidermis was collected 24 hours after the last treatment.

### Mouse model of inhibition of TEWL

Hair was removed from the dorsal skin of mice (shaving followed by depilation), and a latex membrane was applied on the upper half part of the back as described previously (Grubauer et al., 1989; Proksch et al., 1990). The lower half of the back was left uncovered and was used for each mouse as its own control. After 4 days, mice were killed, and the skin was harvested.

### Human subjects

Skin biopsies were taken from nonlesional or lesional (chronic lesions) skin of adult European patients with AD or from lesional skin of adult European patients with psoriasis. Abdominal or breast skin obtained from plastic surgery procedures of adult European subjects was used as healthy control tissue. Human subjects gave skin samples voluntarily after written informed consent. Alternatively, paraffin blocks were used in the frame of a retrospective study. The study was approved by the Ethics Committee of the Medical University of Innsbruck (Innsbruck, Austria) and was conducted in accordance with the Declaration of Helsinki principles (UN4253-297/4.18, UN5073-325/4.2, AN2016-0260-368/4.22-396/5.14 [4117a], EK1412/2220). The number and characteristics of patients are summarized in Table 1.

**Table 1:** Number and Characteristic of Patients Tested

Groups	n	Mean Age ± SEM	Sex	*FLG* genotype	Disease Score
Healthy	6	43.8 ± 5.2	6F	Wild type	
AD	12	50.5 ± 6.8	7F/5M	—	—
Lesional	6	61.7 ± 10.8	3F/3M	1 Heterozygote, 5 ND	ND
Nonlesional	6	39.3 ± 6.0	4F/2M	*FLG* wild type	EASI: 4.8 ± 1.4 (1.0–11.8)
PV	5	54.4 ± 5.3	1F/4M	ND	PASI: 24.6 ± 13.1 (4.1–63.1)

Patient	Details on AD Lesions
1	Lesion on the back
2	Lesion on the abdomen
3	Lesion on the upper breast with impetigo
4	EASI = 9, lesion on the right forearm with impetigo
5	Lesion on the right shoulder, moderate AD
6	EASI = 11.8, lesion on the right forearm, heterozygote for *FLG*

Patient	Details on Psoriatic Lesions
1	PASI = 4.1, lesion on the right thigh, strong erythema, mild inflammation, and scaling
2	Lesion on the right forearm, moderate erythema, inflammation, and scaling
3	PASI = 16.9, lesion on the right thigh, moderate erythema, inflammation, and scaling
4	PASI = 14.4, lesion on the left upper arm, strong erythema, inflammation, moderate scaling
5	PASI = 63.1, lesion on the left upper arm, strong erythema, inflammation, moderate scaling, signs of superinfection

Abbreviations: AD, atopic dermatitis; EASI, eczema area and severity index; F, female; M, male; ND, not determined; PV, psoriasis vulgaris.

### Quantification of gene expression

The epidermis of mouse ears was stored overnight in RNAlater Stabilization Solution (Invitrogen, Carlsbad, CA) at 4 °C. Total RNA was then extracted, and cDNA was prepared according to a previous protocol (Elentner et al., 2018). Primers were purchased from Applied Biosystems (Foster City, CA) (Table 2). TBP and PPIA were used for normalization. Expression fold changes were calculated using the 2^–ΔΔCt^ formula. For pathway-focused gene expression analysis, equal amounts of RNA from each mouse within groups were pooled, and samples were prepared for the PPAR Targets RT^2^ Profiler PCR Array (Qiagen, Hilden, Germany) according to the manufacturer’s instructions. PCR array results for selected genes were validated by qPCR analysis from individual samples. Data are expressed as a heat map, where fold changes of the expression level of genes in a pool of three mice are shown and normalized to the values of control mice. The data shown represent a subset of the entire 89 genes featured in the array, selected for genes involved in FA metabolism. The full gene list is provided in Table 3.

**Table 2:** Taqman Primers used for qPCR

Gene Symbol	Gene Name	Assay Number
*huACOX1*	Human Acyl-CoA-oxidase 1	Hs01074241_m1
*huCXCL8*	Human C-X-C motif chemokine ligand 8	Hs00174103_m1
*huIL18*	Human IL-18	Hs01038788_m1
*huIL1A*	Human IL-1 alpha	Hs00174092_m1
*huIL1B*	Human IL-1 beta	Hs01555410_m1
*huIL33*	Human IL-33	Hs04931857_m1
*huPPIA*	Human Peptidylpropyl isomerase A	Hs04194521_s1
*huS100A8*	Human S100 calcium binding protein A8	Hs00374264_g1
*huS100A9*	Human S100 calcium binding protein A9	Hs00610058_m1
*huTBP*	Human TATA-box binding protein	Forward: 5′-3′ CAC GAA CCA CGG CAC TGA Reverse: 5′-3′ TTT TCT TGC TGC CAG TCT Probe: 5′-3′ TGT GCA CAG GAG CCA AGA GTG AAG A
*huTNF*	Human TNF	Hs00174128_m1
*huTSLP*	Human Thymic stromal lymphopoietin	Hs00263639_m1
*muAcad9*	Mouse Acyl-CoA dehydrogenase family member 9	Mm00554428_m1
*muAcadvl*	Mouse Acyl-Coenzyme A dehydrogenase, very long chain	Mm00444293_m1
*muAcot5*	Mouse Acyl-CoA thioesterase 5	Mm01703166_m1
*muAcot8*	Mouse Acyl-CoA thioesterase 8	Mm00504682_m1
*muAcox1*	Mouse Acyl-CoA-oxidase 1	Mm01246834_m1
*muCpt1a*	Mouse Carnitine palmitoyltransferase 1a	Mm01231183_m1
*muCrot*	Mouse Carnitine O-octanoyltransferase	Mm00470079_m1
*muElovl1*	Mouse Elongation of very long chain fatty acids protein 1	Mm01188316_g1
*muElovl4*	Mouse Elongation of very long chain fatty acids protein 4	Mm00521704_m1
*muElovl6*	Mouse Elongation of very long chain fatty acids protein 6	Mm00851223_s1
*muFasn*	Mouse Fatty acid synthase	Mm00662319_m1
*muG6pdx*	Mouse Glucose-6-phosphate dehydrogenase X-linked	Mm00656735_g1
*muGpi1*	Mouse Glucose phosphate isomerase 1	Mm01962484_u1
*muHk1*	Mouse Hexokinase 1	Mm00439344_m1
*muHk2*	Mouse Hexokinase 2	Mm00443385_m1
*muHsd17b4*	Mouse Hydroxysteroid (17-beta) dehydrogenase 4	Mm00500443_m1
*muIl13*	Mouse IL-13	Mm00434204_m1
*muIl1a*	Mouse IL-1 alpha	Mm00439620_m1
*muIl1b*	Mouse IL-1 beta	Mm00434228_m1
*muK16*	Mouse Keratin 16	Mm01306670_g1
*muLdha*	Mouse Lactate dehydrogenase A	Mm01612132_g1
*muMpc1*	Mouse Mitochondrial pyruvate carrier 1	Mm01316203_g1
*muMpc2*	Mouse Mitochondrial pyruvate carrier 2	Mm00770996_g1
*muPdk1*	Mouse Pyruvate dehydrogenase kinase, isoenzyme 1	Mm00554300_m1
*muPgd*	Mouse Phosphogluconate dehydrogenase	Mm00503037_m1
*muPkm*	Mouse Pyruvate kinase	Mm00834102_gH
*muPpara*	Mouse Peroxisome proliferator-activated receptor alpha	Mm00440939_m1
*muPpard*	Mouse Peroxisome proliferator-activated receptor delta	Mm00803184_m1
*muPparg*	Mouse Peroxisome proliferator-activated receptor gamma	Mm00440940_m1
*muPpia*	Mouse Peptidylpropyl isomerase A	Mm02342430_g1
*muSlc2a1*	Mouse Solute carrier family 2 (facilitated glucose transporter), member 1	Mm00441480_m1
*muSlc2a2*	Mouse Solute carrier family 2 (facilitated glucose transporter), member 2	Mm00446229_m1
*muSlc2a3*	Mouse Solute carrier family 2 (facilitated glucose transporter), member 3	Mm00441483_m1

Abbreviation: CoA, coenzyme A.

**Table 3:** PCR Array Gene List and Respective Fold Changes

Gene	Description	Fold Change (*ft/ft*/CTRL)	Fold Change (*Flg-KO*/CTRL)
*Acaa2*	Acetyl-CoA Acyltransferase 2	0.58	0.78
*Acadl*	Acyl-Coenzyme A dehydrogenase, very long chain	1.33	1.11
*Acadm*	Acyl-Coenzyme A dehydrogenase, medium-chain	1.00	0.91
*Acox1*	Acyl-CoA-oxidase 1	1.54	1.17
*Acox3*	Acyl-CoA-oxidase 3	0.96	0.81
*Acsl1*	Long-chain-fatty-acid-CoA ligase 1	1.21	0.70
*Acsl3*	Long-chain-fatty-acid-CoA ligase 3	0.51	0.87
*Acsl4*	Long-chain-fatty-acid-CoA ligase 4	0.37	0.91
*Acsl5*	Long-chain-fatty-acid-CoA ligase 5	0.51	0.73
*Adipoq*	Adiponectin	0.27	1.28
*Angptl4*	Angiopoietin Like 4	0.20	0.85
*Apoa1*	Apolipoprotein A-I	0.28	0.52
*Apoa5*	Apolipoprotein A-V	10.80	17.62
*Apoc3*	Apolipoprotein C-III	0.13	1.56
*Apoe*	Apolipoprotein E	0.44	0.86
*BC006779*	cDNA sequence BC006779	0.54	0.52
*Cd36*	Fatty acid translocase	0.32	0.83
*Chd9*	Chromodomain helicase DNA binding protein 9	0.72	1.05
*Clu*	Clusterin	1.48	1.04
*Cpt1a*	Carnitine O-palmitoyltransferase 1a	0.61	1.04
*Cpt1b*	Carnitine O-palmitoyltransferase 1b	0.43	0.37
*Cpt2*	Carnitine O-palmitoyltransferase 2	0.94	0.71
*Creb1*	CAMP responsive element binding protein 1	0.98	1.33
*Crebbp*	CREB binding protein	0.86	1.16
*Cyp27a1*	Cytochrome P450, family 27, subfamily a, polypeptide 1	0.62	0.88
*Cyp4a10*	Cytochrome P450, family 4, subfamily a, polypeptide 10	0.17	0.71
*Cyp7a1*	Cytochrome P450, family 7, subfamily a, polypeptide 1	2.27	0.10
*Dgat1*	Diacylglycerol O-acyltransferase 1	0.52	0.80
*Ech1*	Delta(3,5)-Delta(2,4)-dienoyl-CoA isomerase, mitochondrial	0.83	0.75
*Ehhadh*	Peroxisomal bifunctional enzyme	0.23	0.71
*Eln*	Elastin	0.70	0.65
*Ep300*	E1A binding protein p300	0.77	1.02
*Etfdh*	Electron transferring flavoprotein, dehydrogenase	0.93	0.94
*Fabp1*	Fatty acid binding protein 1	0.28	0.22
*Fabp2*	Fatty acid binding protein 2	0.53	0.62
*Fabp3*	Fatty acid binding protein 3	0.27	0.44
*Fabp4*	Fatty acid binding protein 4	0.71	1.21
*Fabp5*	Fatty acid binding protein 5	5.70	1.61
*Fabp6*	Fatty acid binding protein 6	1.02	1.00
*Fabp7*	Fatty acid binding protein 7	141.68	1.24
*Fads2*	Fatty acid desaturase	1.27	1.26
*Fgr*	Gardner-Rasheed feline sarcoma viral (FGR) oncogene homolog	0.38	0.31
*Gyk*	Glycerol kinase	6.26	1.59
*Hif1a*	Hypoxia inducible factor 1, alpha subunit	1.36	1.17
*Hmgcs2*	3-hydroxy-3-methylglutaryl-Coenzyme A synthase 2	0.19	0.90
*Hspd1*	Heat shock protein 1 (chaperonin)	0.76	0.87
*Ilk*	Integrin linked kinase	0.24	0.58
*Klf10*	Kruppel-like factor 10	1.77	1.26
*Lpl*	Lipoprotein lipase	0.86	1.25
*Med1*	Mediator complex subunit 1	0.81	0.99
*Mlycd*	Malonyl-CoA decarboxylase	0.31	0.77
*Mmp9*	Matrix metallopeptidase 9	0.72	0.70
*Ncoa3*	Nuclear receptor coactivator 3	0.58	0.40
*Ncoa6*	Nuclear receptor coactivator 6	1.05	1.17
*Nr1h3*	Nuclear receptor subfamily 1, group H, member 3	0.62	1.19
*Olr1*	Oxidized low density lipoprotein (lectin-like) receptor 1	5.05	0.86
*Pck1*	Phosphoenolpyruvate carboxykinase 1, cytosolic	0.97	1.68
*Pck2*	Phosphoenolpyruvate carboxykinase 2 (mitochondrial)	0.24	0.70
*Pdpk1*	3-Phosphoinositide dependent protein kinase 1	0.85	1.14
*Pltp*	Phospholipid transfer protein	0.41	0.81
*Ppara*	Peroxisome proliferator-activated receptor alpha	0.24	0.88
*Ppard*	Peroxisome proliferator-activated receptor delta	2.04	0.85
*Pparg*	Peroxisome proliferator-activated receptor gamma	0.27	0.90
*Ppargc1a*	Peroxisome proliferative activated receptor, gamma, coactivator 1 alpha	0.37	0.39
*Ppargc1b*	Peroxisome proliferative activated receptor, gamma, coactivator 1 beta	1.26	0.91
*Pprc1*	Peroxisome proliferative activated receptor, gamma, coactivator-related 1	1.22	1.22
*Pten*	Phosphatase and tensin homolog	1.09	1.09
*Rxra*	Retinoid X receptor alpha	0.78	0.97
*Rxrb*	Retinoid X receptor beta	0.70	1.01
*Rxrg*	Retinoid X receptor gamma	0.28	0.40
*Scd1*	Stearoyl-Coenzyme A desaturase 1	0.36	1.22
*Sirt1*	Sirtuin 1	0.63	0.92
*Slc22a5*	Solute carrier family 22 (organic cation transporter), member 5	0.49	0.63
*Slc27a1*	Solute carrier family 27 (fatty acid transporter), member 1	0.66	1.17
*Slc27a2*	Solute carrier family 27 (fatty acid transporter), member 2	0.13	0.28
*Slc27a4*	Solute carrier family 27 (fatty acid transporter), member 4	1.49	1.11
*Slc27a5*	Solute carrier family 27 (fatty acid transporter), member 5	0.47	0.46
*Slc27a6*	Solute carrier family 27 (fatty acid transporter), member 6	1.32	0.90
*Smarcd3*	SWI/SNF related, matrix associated, actin-dependent regulator of chromatin, subfamily d, member 3	0.16	0.37
*Sorbs1*	Sorbin and SH3 domain containing 1	0.90	0.99
*Src*	Rous sarcoma oncogene	0.69	1.37
*Tgs1*	Trimethylguanosine synthase homolog (S. cerevisiae)	0.76	0.93
*Txnip*	Thioredoxin interacting protein	0.29	1.08
*Ucp1*	Uncoupling protein 1 (mitochondrial, proton carrier)	1.00	0.65

Abbreviations: CoA, coenzyme A; CTRL, control; *Flg*-KO, *Flg*-knockout.

### Histological and immunofluorescence analysis

Mouse ears and HEEs were fixed in 4% formaldehyde and embedded in paraffin. Sections of 3 μm thickness were obtained and stained with H&E for routine histology. Immunostaining with anti‒Ki-67 antibody (Roche, Basel, Switzerland) was used to evaluate KC proliferation. Analysis was performed using an Olympus BH-2 light microscope (Olympus, Tokyo, Japan) equipped with a ProgRes C10plus camera and ProgRes CapturePro 2.8.8 image analysis software (Jenoptik, Jena, Germany). For immunofluorescence, sections (3 μm) were deparaffinized, and antigen retrieval was facilitated using 10 mM citrate buffer containing 0.5% Tween (pH 6.0). Sections were blocked with 10% goat serum and thereafter incubated with primary antibodies in 2% goat serum, washed, and incubated with Alexa Fluor 488 goat anti-rabbit secondary antibody (Invitrogen) in 2% BSA. Nuclei were counterstained with DAPI (Invitrogen). Images were acquired with a spinning disk confocal system (UltraVIEW VoX; Perkin Elmer, Waltham, MA) connected to a Zeiss Axio Observer Z1 microscope (Zeiss, Oberkochen, Germany). To determine nuclear localization, nuclei were counterstained with DAPI (Invitrogen), and images were analyzed using GNU Octave software. Channels were merged, and mean fluorescence intensities of the green stain were assigned to gray levels in a gray scale in areas where the green channel exhibited overlap with DAPI nuclear stain. Mean intensities are represented by the spectrum of pseudocolors, ascending from black to white.

### Western blot

For western blot analysis, epidermal sheets or HEEs were lysed in modified RIPA lysis buffer (10 mM Tris-hydrochloride, 150 mM sodium chloride, 1 mM EDTA, 1% Triton X-100, 1% sodium deoxycholate, 0.1% SDS, pH 7.4) in the presence of protease and phosphatase inhibitors (cOmplete Mini and PhosSTOP; Roche). Membranes were incubated overnight with primary antibodies, and bands were detected with Alexa Fluor680 goat anti-rabbit (Invitrogen) or IRDye 800CW goat anti-mouse (Li-COR Biosciences, Lincoln, NE) secondary antibodies. Blots were scanned with an LI-COR Biosciences analyzer. β-Actin was used as a loading control.

### Antibodies

The following primary antibodies were used for western blots and immunofluorescence staining: anti-ACOX1 (ab184032) and anti‒β-actin (ab8227) from Abcam (Cambridge, United Kingdom), anti-ELOVL1 (PA5-39159), anti-GLUT1 (RB-9052-P0) from Thermo Fisher Scientific (Waltham, MA), anti-HK1 (#2024) and pyruvate kinase 1/2 (#3190) from Cell Signaling Technology (Danvers, MA), and anti-FABP5 (12348-1-AP) from Proteintech Group (Rosemont, IL).

### Plasmid construction of lentiviral ACOX1 vector

The lentiviral vector containing human ACOX1 (NM_004035) was generated by cloning human ACOX1 cDNA from the pCMV-ACOX1 (OriGene Technologies, Rockville, MD) plasmid into the BamHI/NotI site of the lentiviral pHR-SIN-CSGW vector (kindly provided by Mary Collins, University College London, London, United Kingdom), thereby generating pHR-SFFV-hACOX1. Sequence-verified lentiviral plasmid was used to generate lentiviral particles. The promoter is a spleen focus forming virus strain P long terminal repeat sequence (SFFV-U3LTR). The original vector has been described in the study by Demaison et al. (2002).

### Generation of lentiviral particles and transduction of KCs

Lentiviral transduction of target cells was performed as described previously (Sigl et al., 2014). In brief, confluent HEK293T cells were transfected with a 1.5 μg lentiviral vector (pHR-SFFV-hACOX1 or pHR-SFFV-Puro) and 0.9 μg of each packaging plasmid (pSPAX2, pMD-G VSV-G, both vectors were kindly provided by D. Trono [École Polytechnique fédérale de Lausanne, Switzerland]) by calcium phosphate‒based transfection. Virus-containing cell supernatant was harvested after 48 hours, filtered through a 0.2 μm filter, diluted at 1:2 with fresh CnT-basal KC medium (CnT-BM.1) supplemented with human KC growth supplements (CnT-07) (CELLnTEC Advanced Cell Systems, Bern, Switzerland), and added to passage 2 KCs in the presence of 1 μg/ml polybrene.

### Generation of HEEs

To generate HEEs, primary human KCs were trypsinized 72 hours after infection and seeded at a density of 3.4 × 10^5^ on inserts with a pore size of 0.4 μm (Merck Millipore, Burlington, MA) in CnT-basal KC medium (CnT-BM.1) supplemented with human KC growth supplements (CnT-07) (CELLnTEC Advanced Cell Systems) (Blunder et al., 2017). After 48 hours, the medium was switched to a mixture of CnT-PR-3D medium (CELLnTEC Advanced Cell Systems) and DMEM (Lonza, Basel, Switzerland) medium (60:40 [v/v]) according to the procedure by Smits et al. (2020). After 16 hours, HEEs were lifted to the air‒liquid interface and cultivated at a humidity of 55–60% at 37 °C and 5% carbon dioxide for an additional 8 days until harvesting. All cell cultures were grown in the absence of antibiotics and antifungals.

### Transmission electron microscopy

Punch biopsies of 3 mm diameter were taken from mouse ears or from HEEs and fixed in Karnovsky’s buffer for 1 hour at room temperature, stored overnight at 4 °C, and then rinsed twice in 0.1 M cacodylate buffer. Thereafter, samples were processed using osmium tetroxide postfixation staining and visualized with a Zeiss 10A (Carl Zeiss, Jena, Germany) or a Hitachi HT 7700 (Tokyo, Japan) electron microscope as described previously (Gruber et al., 2011).

### Quantification of LB numbers and secretion area

LB density was defined as the number of LBs counted per unit area (μm^2^) of the cytoplasm of granular KCs. To quantify secretion areas, the area of secreted content (in nm^2^) was measured over the length of the stratum granulosum‒SC interface (in μm). All image quantifications were performed using ImageJ software.

### Measurement of transepithelial electrical resistance

Transepithelial electrical resistance was measured on HEEs on day 10 with an EVOM3 device (World Precision Instruments, Sarasota, FL) according to the manufacturer’s instruction. Briefly, measurements were performed using 500 μl of a mixture of fresh CnT-PR-3D medium (CELLnTEC Advanced Cell Systems) and DMEM (Lonza) medium (60:40 [v/v]) on top of the inserts and 1,500 μl below the inserts. An insert not seeded with cells was used as a blank. Results are expressed as Ohm/cm^2^.

### Lucifer yellow permeability assay

Penetration of Lucifer yellow was performed as described previously (Blunder et al., 2017). Briefly, 200 μl of 1 mM Lucifer yellow (Sigma-Aldrich) was applied onto HEEs and incubated for 2 hours at 37 °C. Then, HEEs were rinsed with PBS, fixed in formaldehyde, and embedded in paraffin. Thereafter, 6 μm sections were cut, deparaffinized, counterstained with DAPI, and analyzed with an Olympus BX60 epifluorescence microscope (Olympus).

### Determination of TEWL

TEWL was measured with a device from Courage + Khasaka (MP6, Cologne, Germany). Measurements were carried out under light ketamine anesthesia and repeated three times on each ear. The mean values of both ears were used for analysis. Temperature and relative humidity were controlled during the experiment.

### Measurement of epidermal thickness

Epidermal thickness was evaluated on H&E-stained tissue sections using ProgRes CapturePro 2.8.8 image analysis software (Jenoptik). For each mouse, serial measurements were performed at 100 μm intervals on three different areas (from the stratum basale to the stratum granulosum).

### Epidermal lipid analysis

Epidermal lipids were extracted from snap-frozen epidermis obtained by trypsinization from the dorsal skin of mice. Samples were weighed and extracted according to the procedure used by Matyash et al. (2008) in 700 μl methyl-tert-butyl ether/methanol (3/1, v/v) containing 500 pmol butylated hydroxytoluene, 1% acetic acid, and internal standards (1.4 nmol of FA 17:0 and FA 21:0; 400 pmol of d18:1/17:0 Cer, Avanti Polar Lipids, Alabaster, AL). Total lipid extraction was performed under constant shaking for 30 minutes at room temperature. After the addition of 140 μl H_2_O and further incubation for 30 minutes at room temperature, samples were centrifuged at 1,000*g* for 10 minutes. A total of 500 μl of the upper, organic phase was collected and dried under a stream of nitrogen. Lipids were resolved in 500 μl 2-propanol/methanol/H_2_O (7/2.5/1, v/v/v) for ultra-HPLC-quadrupole time-of-flight mass spectrometry analysis. A total of 50 μl of resolved lipids were used for the derivatization of FAs using the AMP + MaxSpec Kit (Cayman Chemical, Ann Abor, MI) according to the manufacturer’s instructions. The remaining epidermal sheets were solubilized in 0.3N sodium hydroxide at 65 °C for 4 hours, and the protein content was determined using Pierce BCA reagent (Thermo Fisher Scientific) and BSA as a standard.

Chromatographic separation was performed using an AQUITY-UPLC system (Waters Milford, MA) as described (Knittelfelder et al., 2014) with two modifications: (i) a Luna ω-C18 column (2.1 × 50 mm, 1.6 μm; Phenomenex, Torrance, CA) was employed and (ii) a 20-minute linear gradient was started with 80% solvent A (methanol/H_2_O, 1/1, v/v; 10 mM ammonium acetate, 0.1% formic acid, 8 μM phosphoric acid). A SYNAPTG1 qTOF HD mass spectrometer (Waters) equipped with an electrospray ionization source was used for the detection of lipids in positive ionization mode. Data were acquired using MassLynx 4.1 Software (Waters). Lipids were manually identified and analyzed with Lipid Data Analyzer 2.5.3 software (Hartler et al., 2011). Data were normalized for recovery, extraction, and ionization efficacy by calculating analyte–to–internal standard ratios (arbitrary unit) and expressed as AU/mg protein.

### Measurement of intracellular ATP content

ATP content in epidermal cell pellets was quantified using the ATPlite 1step (Perkin Elmer) assay kit. Luminescence was read with a Tecan Infinite Pro 200 reader. Values were normalized to total protein content.

### Measurement of glucose, pyruvate, and lactate concentration

Punch biopsies taken from the dorsal skin of the mice were trypsinized, and the obtained epidermal sheets were incubated in 250 μl CnT-Prime 3D Barrier culture medium (CELLnTEC Advanced Cell Systems) in duplicate at 37 °C and 5% carbon dioxide. After 24 hours, the medium was collected, deproteinized using a 10 kDa MWCO spin filter, and assayed for glucose and pyruvate concentration using a colorimetric assay kit (Sigma-Aldrich). The concentration of lactate in the medium was evaluated as previously described (Limonciel et al., 2011). Fresh medium was used in all the assays as a reference. Results were normalized by the total protein content of the tissue.

### Measurement of acyl-CoA oxidase activity

Epidermal cell suspensions from mouse ears were prepared as reported earlier (Elentner et al., 2018). Cells were counted, pelleted, and frozen in liquid nitrogen. On the day of analysis, cell pellets were resuspended in Buffer A (250 mM sucrose, 20 mM Tris-hydrochloride, 2 mM EDTA, 1 mg/ml BSA, pH 7.2). To promote membrane permeabilization, samples were subjected to one cycle of snap freezing/thawing. Thereafter, mitochondria and peroxisomes were sedimented by centrifugation at 13,000*g* for 1.5 minutes at 4 °C. Pellets enriched in mitochondria and peroxisomes were resuspended in Buffer A. The efficacy of organelle enrichment was controlled by western blot analysis. The activity of acyl-CoA oxidase was measured by a fluorometric-based method with some modifications (Vamecq, 1990). Briefly, the assay medium contained 50 mM Tris-hydrochloride buffer (pH 8.0), 1 U/ml horseradish peroxidase type II (Sigma-Aldrich), 2 μM BSA, 3 μM flavin-adenin-dinukleotid (Sigma-Aldrich), and 40 mM aminotriazole (Sigma-Aldrich) to inhibit endogenous catalase. To monitor hydrogen peroxide generation, homovanillic acid was replaced by 5 μM Amplex Red (Thermo Fisher Scientific), which is more sensitive and reacts with hydrogen peroxide with a 1:1 stoichiometry to produce resorufin. About 15–20 μg of protein were preincubated with assay medium for 3 minutes at 37 °C. The reaction was initiated by the addition of 500 μM CoA esters (lignoceroyl- and hexacosanoyl-CoA from Avanti Polar Lipids), and the increase in fluorescence was recorded for 20 minutes at 37 °C, ex = 563 nm and em = 595 nm. Results were normalized by the protein content of the cell homogenates.

### Measurements of activity of respiratory complexes

Complex I activity was measured using a dichlorophenolindophenol-coupled method (Angebault et al., 2011). In brief, cellular fractions were preincubated with assay medium containing 80 mM potassium phosphate (pH 7.4), 1 mM potassium cyanide, 2 mM sodium azide, 0.075 mM dichlorophenolindophenol, and 0.1 mM decylubiquinone. Thereafter, 0.3 mM NADH was added, and the decrease in absorbance was recorded at 600 nm. Complex II activity was measured according to the methods by Rustin et al. (1994)). In brief, assay medium contained 100 mM potassium phosphate (pH 7.5), 0.5 M succinate, 10 mM potassium cyanide, 20 mM phenazine, and 500 μM dichlorophenolindophenol. Succinate-dependent reduction of dichlorophenolindophenol was measured at 600 nm.

### Measurement of long-chain acyl-CoA dehydrogenase activity

Dehydrogenation of palmitoyl-CoA mediated by mitochondrial dehydrogenases was measured according to the methods by Lehman et al. (1990). Briefly, cellular fractions were preincubated in 100 mM potassium phosphate buffer (pH 7.2) containing 0.2% Triton X-100, 0.1 mM EDTA, and 150 μm ferricenium ion. The reaction was initiated by the addition of 200 μM palmitoyl-CoA, and the decrease in absorbance at 300 nm was recorded for 5 minutes.

### Liquid chromatography‒mass spectrometry‒based metabolomic profiling

Tissue samples were homogenized using a Precellys 24 tissue homogenizer (Precellys CK14 lysing kit, Bertin Technologies, Rockville, MD). A total of 9 μl of methanol was added per mg tissue. A total of 30 μl of the homogenized tissue samples was transferred into a glass vial, and 20 μl of metabolite internal standard mix and 270 μl methanol were added. After vortexing, the mixture was incubated in a shaker for 20 minutes on ice. The samples were centrifuged at 500*g* for 10 minutes. The supernatant was collected and dried using a nitrogen evaporator. Afterward, samples were reconstituted in 100 μl water and were used for the metabolite analysis. A 1290 Infinity II UHPLC system (Agilent Technologies, Santa Clara, CA) coupled with a 6470 triple quadrupole mass spectrometer (Agilent Technologies) was used for the liquid chromatography with tandem mass spectrometry analysis. The chromatographic separation for samples was carried out on a ZORBAX RRHD Extend-C18, 2.1 × 150 mm, 1.8 μm analytical column (Agilent Technologies). The column was maintained at a temperature of 40 °C, and 4 μl of the sample was injected per run. The mobile phase A was 3% methanol (v/v), 10 mM tributylamine, and 15 mM acetic acid in water, and the mobile phase B was 10 mM tributylamine and 15 mM acetic acid in methanol. The gradient elution with a flow rate of 0.25 ml/minute was performed for a total time of 24 minutes. Afterward, a backflushing of the column using a 6port/2-position divert valve was carried out for 8 minutes using acetonitrile, followed by 8 minutes of column equilibration with 100% mobile phase A. The triple quadrupole mass spectrometer was operated in an electrospray ionization negative mode, spray voltage of 2 kV, gas temperature of 150 °C, gas flow of 1.3 l/min, nebulizer 45 psi, sheath gas temperature of 325 °C, and sheath gas flow of 12 l/min. The metabolites of interest were detected using a dynamic MRM mode. The MassHunter 10.0 software (Agilent Technologies) was used for the data processing. Ten-point linear calibration curves with internal standardization were constructed for the quantification of metabolites. Metabolite concentrations were expressed as pmol/mg tissue. The full list of the quantified metabolites and respective fold changes are provided in Table 4.

**Table 4:** Intracellular Metabolites Quantified by LC‒MS in Mouse Epidermis and Respective Fold Changes

Metabolite	Fold Change (*ft/ft*/CTRL)	Fold Change (*Flg-*KO/CTRL)
Arginine	0.15	0.36
Histidine	0.36	0.45
Thiamine	0.99	0.97
Carnitine	0.79	0.48
Asparagine	0.89	0.61
Serine	0.19	0.38
Cystathionine	0.17	0.83
Glycine	0.22	0.40
Glutamine	0.58	0.46
Cystine	0.52	0.46
Hydroxy-proline	0.88	0.34
Taurine	1.92	0.86
Hexose	1.33	0.71
Pentose	0.99	0.53
Citrulline	1.09	1.18
Threonine	1.04	0.65
Arabitol	0.80	0.49
Xylitol	0.81	0.50
Creatine	1.40	0.57
Allantoin	0.87	0.43
Proline	0.21	0.40
Guanidobutyric acid	0.29	0.44
N-Acetyl-galactosamine	0.82	0.80
Creatinine	0.79	0.44
Acetylcarnitine	0.89	0.68
Deoxyribose	0.76	0.41
Homocystine	0.84	0.81
Valine	0.29	0.41
Uracil	1.40	0.59
O-Phosphorylethanolamine	3.19	1.14
Cytidine	1.21	0.75
Hypoxanthine	0.61	0.61
Methionine	1.67	0.72
Guanine	0.22	0.75
Deoxycytidine	0.44	0.82
Xanthine	0.80	0.59
Isoleucine	1.30	0.74
Tyrosine	0.81	0.67
Uridine	1.34	0.76
Leucine	1.10	0.55
Pyridoxine	0.44	0.39
Deoxyuridine	0.44	0.77
Thymine	0.66	0.44
Inosine	0.77	0.69
Deoxyinosine	0.08	0.80
Guanosine	0.31	0.65
Deoxyguanosine	0.10	0.66
Aminoadipic acid	1.40	0.58
Phenylalanine	1.02	0.61
Glutamic acid	1.03	0.58
Aspartic Acid	1.21	0.69
Hydroxy-glutamic acid	0.43	0.51
S-Adenosyl-homocysteine	1.62	0.75
Thymidine	0.25	0.74
Pentahydroxyhexanoic acid	1.22	0.39
Uric acid	1.25	0.72
Glyceric acid	0.48	0.53
Quinic acid	0.82	1.19
Glucoheptonic acid	0.40	0.31
N-Acetylneuraminic acid	2.38	0.85
Adenosine	0.73	0.80
Oxamic acid	0.50	1.21
Hexose-6-phosphate	5.21	2.20
Dehydroshikimic acid	1.10	1.00
Lactic acid	0.88	0.71
Creatine phosphate	1.81	1.34
Tryptophan	0.97	0.73
Ribose-5-phosphate	1.87	0.95
Sedoheptulose-7-phosphate	1.72	0.79
Hexose 1-phosphate	1.23	1.15
Xanthosine	0.60	0.54
N-Acetyl-glucosamine phosphate	1.05	0.77
Xylulose-5-phosphate	2.42	0.86
Hydroxynicotinic acid	0.55	0.53
Orotic acid	0.73	0.57
Cytidine monophosphate	3.83	1.19
Nicotinamide adenine dinucleotide	1.75	0.98
Pyruvic acid	0.33	0.67
Dihydroxyacetone phosphate	1.29	0.94
Uridine monophosphate	2.51	1.07
Dihydroxyisovalerate	0.96	0.24
Inosine monophosphate	1.94	0.99
Deoxy-(methylthio)adenosine	0.56	0.70
Nicotinic acid	0.16	0.35
Pantothenic acid	1.00	0.58
Adenosine monophosphate	1.83	0.98
Riboflavin	0.57	0.33
Vanillic acid	0.27	0.37
N-Formyl-Y	0.43	0.56
Maleic acid	0.68	0.76
Hydroxy-indoleacetic acid	1.31	0.67
Succinic acid	0.93	0.57
Adenosine cyclic monophosphate	0.70	0.77
Glutathione oxidized	1.16	1.00
N-Carbamoyl-aspartic acid	0.95	0.73
Hydroxybenzoic acid	0.76	0.48
N-Carbamyl-glutamic acid	0.45	0.41
Malic acid	1.69	0.76
Citramalic acid	0.77	0.64
Hydroxyglutaric acid	0.66	0.62
Adipic acid	0.74	1.03
N-Acetylglutamic acid	1.37	0.45
Uridine diphosphohexose	1.96	1.22
Dimethyl Succinic acid	0.81	1.08
alpha-Ketoglutaric acid	0.89	1.10
Dihydroxybenzoic acid	1.88	1.41
Pyridinedicarboxylic acid	0.99	0.86
Phosphoglyceric acid	1.06	0.96
Citric acid + Isocitric acid	1.02	0.60
Pyridoxic acid	1.21	0.33
Aconitic acid	0.96	0.60
Homocitrate	0.90	0.80
Hydroxy hippuric acid	0.71	0.63
Phenylpyruvic acid	1.95	0.83
Palmitoylcarnitine	0.79	0.62

Abbreviations: CTRL, control; *Flg*-KO, *Flg*-knockout; LC‒MS, liquid chromatography–mass spectrometry.

### Assay of protein content

Total protein content was determined using the Bio-Rad reagent protein assay (Bio-Rad Laboratories, Hercules, CA) with BSA as a standard. The absorbance was measured at 595 nm.

### Statistical analysis

Statistical analyses were performed using GraphPad Prism Software 8.0. Results are expressed as mean ± SEM. Statistical significance was determined between groups using a Student’s *t*-test with ∗*P* < 0.05, ∗∗*P* < 0.01, ∗∗∗*P* < 0.001, and ∗∗∗∗*P* < 0.0001 as significant levels. The symbol n represents the cumulated number of mice per group. The experiments were performed multiple times.

### Data availability statement

Microarray dataset related to this article can be found at https://data.mendeley.com/datasets/kpc5m4rv5t/1, hosted at Mendeley data (10.17632/kpc5m4rv5t.1).

## ORCIDs

Petra Pavel: http://orcid.org/0000-0001-6753-614X

Géraldine Leman: http://orcid.org/0000-0002-4500-6700

Martin Hermann: http://orcid.org/0000-0003-2213-3448

Christian Ploner: http://orcid.org/0000-0002-0313-2960

Thomas O. Eichmann: http://orcid.org/0000-0002-8521-2795

Deborah Minzaghi: http://orcid.org/0000-0002-3151-0260

Franz P. W.Radner: http://orcid.org/0000-0003-3466-0181

Barbara Del Frari: http://orcid.org/0000-0002-3089-7109

Robert Gruber: http://orcid.org/0000-0003-3358-1684

Sandrine Dubrac: http://orcid.org/0000-0002-2936-8488

## Author Contributions

Conceptualization: SD, PP; Data Curation: SD, PP, GL, CP, TOE, FPWR, MH; Formal Analysis: SD, PP, GL, CP, TOE, FPWR, MH; Funding Acquisition: SD; Investigation: SD, PP, GL, CP, TOE, FPWR, DM, MH; Methodology: SD, PP, GL, CP, TOE, FPWR; Project administration: SD, PP; Resources: BDF, RG, PP; Supervision: SD; Validation: SD, PP, GL, TOE, MH; Visualization: SD, PP; Writing - Original Draft Preparation: SD, PP; Writing - Review and Editing: PP, GL, MH, CP, TOE, DM, FPWR, BDF, RG, SD
